# 24-Norursodeoxycholic acid ameliorates experimental alcohol-related liver disease and activates hepatic PPARγ

**DOI:** 10.1016/j.jhepr.2023.100872

**Published:** 2023-08-03

**Authors:** Christoph Grander, Moritz Meyer, Daniel Steinacher, Thierry Claudel, Bela Hausmann, Petra Pjevac, Felix Grabherr, Georg Oberhuber, Manuel Grander, Natascha Brigo, Almina Jukic, Julian Schwärzler, Günter Weiss, Timon E. Adolph, Michael Trauner, Herbert Tilg

**Affiliations:** 1 Department of Internal Medicine I, Gastroenterology, Hepatology, Endocrinology and Metabolism, Medical University Innsbruck, Innsbruck, Austria; 2 Hans Popper Laboratory of Molecular Hepatology, Department of Internal Medicine III, Division of Gastroenterology and Hepatology, Medical University of Vienna, Vienna, Austria; 3 Joint Microbiome Facility of the Medical University of Vienna, The University of Vienna, Vienna, Austria; 4 Department of Laboratory Medicine, Medical University of Vienna, Vienna, Austria; 5 Division of Microbial Ecology, Department of Microbiology and Ecosystem Science, Centre for Microbiology and Environmental Systems Science, University of Vienna, Vienna, Austria; 6 INNPATH, Tirol-Kliniken University Hospital Innsbruck, Innsbruck, Austria; 7 Department of Internal Medicine II, Infectious Diseases, Immunology, Rheumatology, Pneumology, Medical University Innsbruck, Innsbruck, Austria

**Keywords:** Alcoholic liver disease, norUDCA, Microbiota, M2 macrophages, Ppar-gamma

## Abstract

**Background & Aims:**

Alcohol-related liver disease (ALD) is a global healthcare challenge with limited treatment options. 24-Norursodeoxycholic acid (NorUDCA) is a synthetic bile acid with anti-inflammatory properties in experimental and human cholestatic liver diseases. In the present study, we explored the efficacy of norUDCA in experimental ALD.

**Methods:**

NorUDCA was tested in a preventive and therapeutic setting in an experimental ALD model (Lieber–DeCarli diet enriched with ethanol). Liver disease was phenotypically evaluated using histology and biochemical methods, and anti-inflammatory properties and peroxisome proliferator-activated receptor gamma activation by norUDCA were evaluated in cellular model systems.

**Results:**

NorUDCA administration ameliorated ethanol-induced liver injury, reduced hepatocyte death, and reduced the expression of hepatic pro-inflammatory cytokines including *tumour necrosis factor* (*Tnf*), *Il-1β*, *Il-6*, and *Il-10*. NorUDCA shifted hepatic macrophages towards an anti-inflammatory M2 phenotype. Further, norUDCA administration altered the composition of the intestinal microbiota, specifically increasing the abundance of *Roseburia*, *Enterobacteriaceae*, and *Clostridum* spp. In a therapeutic model, norUDCA also ameliorated ethanol-induced liver injury. Moreover, norUDCA suppressed lipopolysaccharide-induced IL-6 expression in human peripheral blood mononuclear cells and evoked peroxisome proliferator-activated receptor gamma activation.

**Conclusions:**

NorUDCA ameliorated experimental ALD, protected against hepatic inflammation, and affected gut microbial commensalism. NorUDCA could serve as a novel therapeutic agent in the future management of patients with ALD.

**Impact and implications:**

Alcohol-related liver disease is a global healthcare concern with limited treatment options. 24-Norursodeoxycholic acid (NorUDCA) is a modified bile acid, which was proven to be effective in human cholestatic liver diseases. In the present study, we found a protective effect of norUDCA in experimental alcoholic liver disease. For patients with ALD, norUDCA could be a potential new treatment option.

## Highlights

• Alcohol-related liver disease is a global healthcare concern with limited treatment options.
• NorUDCA is a modified bile acid which was proven to be effective in human cholestatic liver diseases.
• We observed an anti-inflammatory effect of norUDCA in experimental alcohol-related liver disease.
• NorUDCA treatment results in a shift towards an M2 macrophage type.

## Graphical abstract

**Figure:**
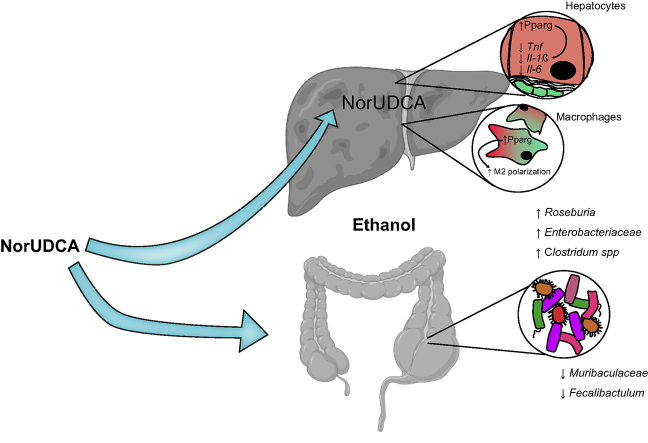

## Introduction

Alcohol overconsumption is estimated to cause 5.3% of all deaths worldwide^1^^,^^2^ and is still the most common indication for liver transplantation in Europe.^3^ The hepatic manifestation of alcohol overconsumption is alcohol-related liver disease (ALD), comprising a spectrum from hepatic steatosis, hepatitis (alcoholic hepatitis) to fibrosis, cirrhosis, and hepatocellular carcinoma. The pathogenesis of ALD is multilayered and involves hepatic ethanol toxicity resulting in the generation of reactive oxygen species,^4^ as well as the increased translocation of pathogen-associated molecular patterns (such as lipopolysaccharide [LPS]) from the leaky gut into the systemic circulation. Ethanol consumption has been shown to alter the intestinal microbiota, leading to a decrease in gut barrier function.[5], [6], [7], [8] Furthermore, alcohol consumption perturbates bile acid metabolism.^9^^,^^10^ For example, ethanol downregulates farnesoid X receptor (FXR/NR1H4),^11^^,^^12^ alters bile acid conjugation,^13^ and affects enterohepatic bile acid circulation.^13^^,^^14^ Chronic alcohol consumption results in an increased bile acid pool and decreased excretion of bile acids. Increased exposure to toxic bile acids might thereby aggravate hepatic injury.^14^ All these factors facilitate the production of pro-inflammatory cytokines such as IL-6, tumour necrosis factor (TNF), and IL-1β along with the infiltration of pro-inflammatory cells,^15^ which culminates in cell necrosis, liver injury, and fibrogenesis.^16^^,^^17^

In addition to inflammatory mediators, numerous immune cell populations are involved in the pathogenesis of ALD. Studies have shown that infiltrating macrophages polarise in response to microenvironmental signals and play a crucial role in pathophysiological processes such as inflammation, tumour development, tissue repair, and metabolism.^15^^,^[18], [19], [20] Ethanol is known to promote the polarisation of M1 macrophages through NF-κB signalling.^21^ Peroxisome proliferator-activated receptor gamma (PPARg/NR1C3) is a nuclear receptor inhibiting the expression of inflammatory cytokines and inducing the differentiation of immune cells towards an anti-inflammatory (M2) phenotype.[22], [23], [24]

24-Norursodeoxycholic acid (NorUDCA) is a side chain-shortened ursodeoxycholic acid (UDCA) with potent choleretic properties.^25^^,^^26^ Owing to the relative resistance of norUDCA to conjugation, it is reabsorbed by cholangiocytes and shunted between bile ducts and hepatocytes, resulting in increased hepatic exposure and secretion of biliary bicarbonate shielding the bile epithelia from bile acid toxicity.[25], [26], [27] NorUDCA ameliorated the pathology in *Mdr2*-deficient mice, a model of primary sclerosing cholangitis (PSC),^28^^,^^29^ and showed potent anti-inflammatory effects in mouse models of non-cholestatic liver injury such as hepatic schistosomiasis,^28^ experimental non-alcoholic steatohepatitis,^27^ and acute non-cytolytic lymphocytic choriomeningitis virus infection.^30^ Importantly, when compared with placebo, norUDCA was effective in reducing serum levels of alkaline phosphatase and transaminases in patients with PSC.^31^ Furthermore, in a recent placebo-controlled clinical trial,^32^ treatment of patients with non-alcoholic fatty liver disease (NAFLD) with norUDCA resulted in a dose-dependent reduction of serum alanine aminotransferase (ALT) within 12 weeks, suggesting efficacy in human NAFLD.

Here, we explored the therapeutic efficacy of norUDCA in experimental ALD. NorUDCA treatment ameliorated experimental liver disease, as demonstrated by reduced liver injury, reduced hepatocyte death, and reduced expression of pro-inflammatory cytokines. NorUDCA enhanced PPARg activity independent from ethanol exposure and increased abundance of potentially beneficial bacteria.

## Materials and methods

### Mouse experiments

Two different models of experimental ALD were used to study the role of norUDCA in ALD. All experiments were aligned to ethical principles according to Austrian laws (2020-0.152.544) and were carried out in the animal facility of the Medical University of Innsbruck. (1) To study the effect of norUDCA in a preventive setting, 7- to 8-week-old female wild-type (wt/wt; C57BL/6) mice were fed a Lieber–DeCarli diet containing 1–5 vol% (ethanol-fed) *ad libitum* for 15 days (Safe, Rosenberg, Germany). Control groups were fed a pair diet containing isocaloric maltose. Half of the ethanol-fed and pair-fed groups were treated with norUDCA (1 mg/ml diet, 5% wt/wt^30^) by supplementing norUDCA into the diet. (2) To study possible therapeutic effects of norUDCA in experimental ALD, 7- to 8-week-old female wild-type mice were fed a Lieber–DeCarli diet containing 1–5 vol% (ethanol-fed) *ad libitum* for 15 days. NorUDCA treatment was conducted from Day 10 to Day 15.

### Histology

Liver tissue samples were fixed in formaldehyde (Sigma, St. Louis, MO, USA) immediately after sacrifice. Samples were embedded in paraffin and further prepared at the Department of Pathology at the Medical University of Innsbruck. Liver sections were deparaffinised in xylene and dehydrated in an ethanol gradient. Dehydration, embedding, and H&E staining^33^ were carried out at the Department of Pathology at the Medical University of Innsbruck, before stained slides were evaluated by an experienced pathologist (GO, INNPATH). Up to 20 high-power fields (1 mm^2^) per slide were analysed.

### Microbiome studies

The microbial community composition in collected caecal stool samples was analysed by ribosomal small subunit (SSU rRNA/16S rRNA) gene amplicon sequencing^34^ at the Joint Microbiome Facility (Vienna) under the Project ID at the Joint Microbiome Facility (Vienna). DNA extraction from stool samples using the QIAamp DNA Fast stool Kit was automated on the QiaCube Connect. For microbial community profiling, the 16S rRNA genes were amplified by PCR applying primers that cover most bacterial and archaeal clades (515F, 806R).^35^ After PCR amplification of the marker gene region, the amplicons were barcoded, multiplexed, and sequenced on the Illumina MiSeq platform at the Joint Microbiome Facility.^34^ Negative controls were performed during sampling, DNA extraction, and barcoding. Further details on amplicon sequence data processing are provided in the Supplementary methods.

### Data availability

SSU rRNA gene amplicon datasets are deposited in Sequence Read Archive (SRA) under the BioProject accession number PRJNA907184.

### Statistical analysis

For analysing the present data, we used GraphPad Prism 5 (La Jolla, CA, USA). Unpaired two-tailed Student’s *t* test, the Kruskal–Wallis test followed by Dunn’s multiple comparison test, and one-way ANOVA followed by the *post hoc* Newman–Keuls test were used where appropriate. Two or more independent experiments were performed for each modality. Results are shown as mean ± SEM. Statistical significance was considered at *p* <0.05.

Further information on materials and methods are provided in the Supplementary materials.

## Results

### NorUDCA treatment protects from experimental ALD

**Fig. 1:**
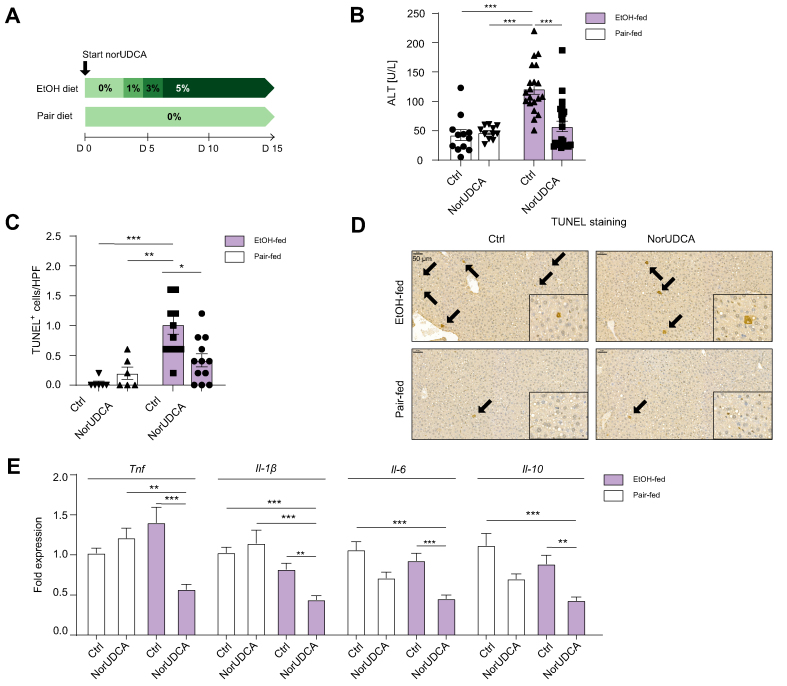
NorUDCA protects from experimental alcoholic liver disease. (A) Graphical illustration of experimental design. (B) Serum concentrations of ALT and (C) number of TUNEL^+^ cells were significantly decreased in norUDCA-treated, EtOH-fed mice compared with Ctrl. (D) Representative images and quantification of TUNEL^+^ liver cells per HPF based on immunoreactivity (brown indicates TUNEL^+^ cells, black arrows indicate positivity of TUNEL+ cells). (E) NorUDCA treatment significantly decreased the mRNA expression of *Tnf*, *Il-1β*, *Il-6*, and *Il-10* in EtOH-fed mice compared with house-keeping gene β-actin. Data are shown as mean ± SEM. ∗*p* <0.05, ∗∗*p* <0.01, and ∗∗∗*p* <0.001 according to one-way ANOVA with Bonferroni *post hoc* analysis or the Kruskal–Wallis test with Dunn’s *post hoc* analysis. β-actin was used as a housekeeping gene (E). ALT, alanine aminotransferase; Ctrl, control; EtOH, ethanol; HPF, high-power field; norUDCA, 24-norursodeoxycholic acid; *Tnf*, tumour necrosis factor; TUNEL^+^, terminal deoxynucleotidyl transferase dUTP nick end labelling-positive.

Mice were fed an ethanol-enriched diet with/without norUDCA (1 mg/ml, 5% wt/wt) supplementation for 15 days (Fig. 1A). Ethanol feeding resulted in liver injury, as indicated by significantly increased ALT levels (*p* <0.001; Fig. 1B) and an increased number of intrahepatic terminal deoxynucleotidyl transferase dUTP nick end labelling-positive (TUNEL^+^) apoptotic hepatocytes, when compared with that in pair-fed control mice (Fig. 1C and D). NorUDCA supplementation reduced ALT levels (*p* <0.001; Fig. 1B) and the number of TUNEL^+^ cells (*p* <0.05; Fig. 1C and D) in ethanol-fed mice. Serum ethanol concentrations were not significantly different between ethanol-fed groups (Fig. S1A), but mRNA expression of ethanol-metabolising enzymes such as alcohol dehydrogenase-1 (*Adh-1*) (*p* <0.01; Fig. S1B), *Adh-5* (*p* <0.001; Fig. 1C), aldehyde dehydrogenase-1a1 (*Aldh-1a1*) (*p* <0.001; Fig. S1D), and *Aldh-4a1* (*p* <0.001; Fig. S1E) was significantly increased in norUDCA-treated mice compared with ethanol-fed controls. To further investigate the influence of norUDCA on ethanol-induced hepatic injury, we analysed the expression of pro-inflammatory cytokines. NorUDCA reduced the hepatic mRNA expression of *Tnf* (*p* <0.001), *Il-1β* (*p* <0.01), *Il-6* (*p* <0.001), and *Il-10* (*p* <0.01) compared with that in ethanol-fed controls (Fig. 1E).

### M2 macrophage polarisation is induced by norUDCA

**Fig. 2:**
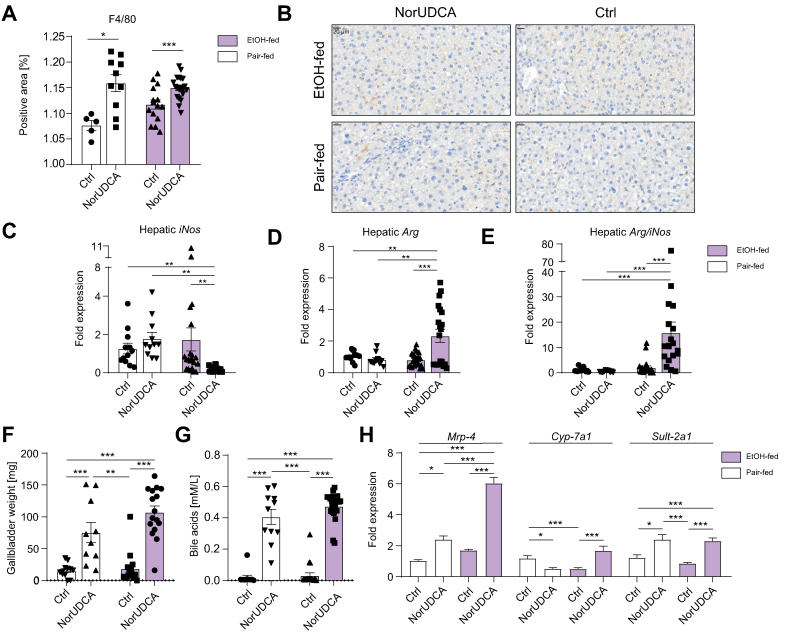
NorUDCA induces a M2 hepatic macrophage phenotype. (A) Quantification of F4/80^+^-positive area per high-power field based on F4/80 immunoreactivity. (B) Representative images of F4/80^+^-stained liver sections (brown indicates F4/80^+^ cells). Pronounced M2 macrophage phenotype could be observed in norUDCA-treated, EtOH-fed mice, indicated by (C) significantly decreased hepatic *iNos* and (D) significantly increased Arg expression as well as (E) *Arg*/*iNos* ratio. (F) Gallbladder weight and (G) serum bile acid concentrations were increased in norUDCA-treated mice in pair-fed and EtOH-fed mice. (H) NorUDCA treatment significantly altered the hepatic expression of proteins involved in bile acid metabolism such as *Mrp4* (left), *Cyp7a1* (middle), and *Sult2a1* (right). Data are shown as mean ± SEM. ∗*p* <0.05, ∗∗*p* <0.01, and ∗∗∗*p* <0.001 according to one-way ANOVA with Bonferroni *post hoc* analysis or the Kruskal–Wallis test with Dunn’s *post hoc* analysis. β-actin was used as a housekeeping gene (C–E, H). *Arg*, arginase; Ctrl, control; *Cyp7a1*, cytochrome P450 family 7 subfamily A member 1; EtOH, ethanol; *iNos*, inducible nitric oxide synthase; *Mrp4*, multidrug resistance-associated protein 4; norUDCA, 24-norursodeoxycholic acid; *Sult2a1*, sulfotransferase family 2A member 1.

In a next step, the number of hepatic macrophages was quantified by F4/80 staining. Ethanol feeding tended to increase the numbers of hepatic F4/80^+^ cells (*p* = 0.104; Fig. 2A and B), whereas norUDCA treatment significantly increased the number of hepatic F4/80^+^ cells in both ethanol-fed (*p* <0.001; Fig. 2A and B) and pair-fed mice (*p* <0.05; Fig. 2A and B). In norUDCA-treated, ethanol-fed mice, hepatic macrophage composition predominantly consisted of anti-inflammatory M2 polarised macrophages, as indicated by a decreased hepatic expression of inducible nitric oxide synthase (*iNos*) (*p* <0.01; Fig. 2C) and an increased expression of arginase (*Arg*) (*p* <0.001; Fig. 2D), *Arg*/*iNos* ratio (*p* <0.001; Fig. 2E), and *Cd206/iNos* ratio (Fig. S2A and B). To further evaluate this finding *in vitro*, we performed FACS analysis of murine bone marrow derived macrophages after stimulation with norUDCA, LPS, IL-4, and interferon gamma.^36^ NorUDCA treatment significantly shifted the relative number of M1/M2 in favour of M2 macrophages compared with that in LPS-stimulated controls (*p* <0.001; Fig. S2C–G). Ethanol feeding resulted in increased infiltration of myeloperoxidase-positive neutrophils (Fig. S2H and I).

### NorUDCA affects bile acid metabolism

As expected, norUDCA treatment altered bile acid metabolism. An increased gallbladder weight could be observed in both pair-fed (*p* <0.001; Fig. 2F) and ethanol-fed mice (*p* <0.001; Fig. 2F) treated with norUDCA, in line with its potent choleretic effects. Similarly, plasma bile acid concentration was significantly higher in norUDCA-treated mice in both the pair-fed (*p* <0.001; Fig. 2G) and ethanol-fed (*p* <0.001; Fig. 2G) groups, whereas no difference in gallbladder weight or bile acid concentration could be observed between control groups (Fig. 2F and G). Furthermore, norUDCA significantly increased the hepatic mRNA expression of enzymes involved in the regulation of bile acid synthesis, metabolism, and export, including multidrug resistance-associated protein 4 *(Mrp4*) (*p* <0.001; Fig. 2H, left panel), cytochrome P450 family 7 subfamily A member 1 (*Cyp7a1*) (*p* <0.001; Fig. 2H, middle panel), sulfotransferase family 2A member 1 *(Sult2a1*) (*p* <0.001; Fig. 2H, right panel), and acyl-CoA oxidase 1 *(Acox1*) (*p* <0.001; Fig. S1F).

### NorUDCA impacts on hepatic lipid metabolism

**Fig. 3:**
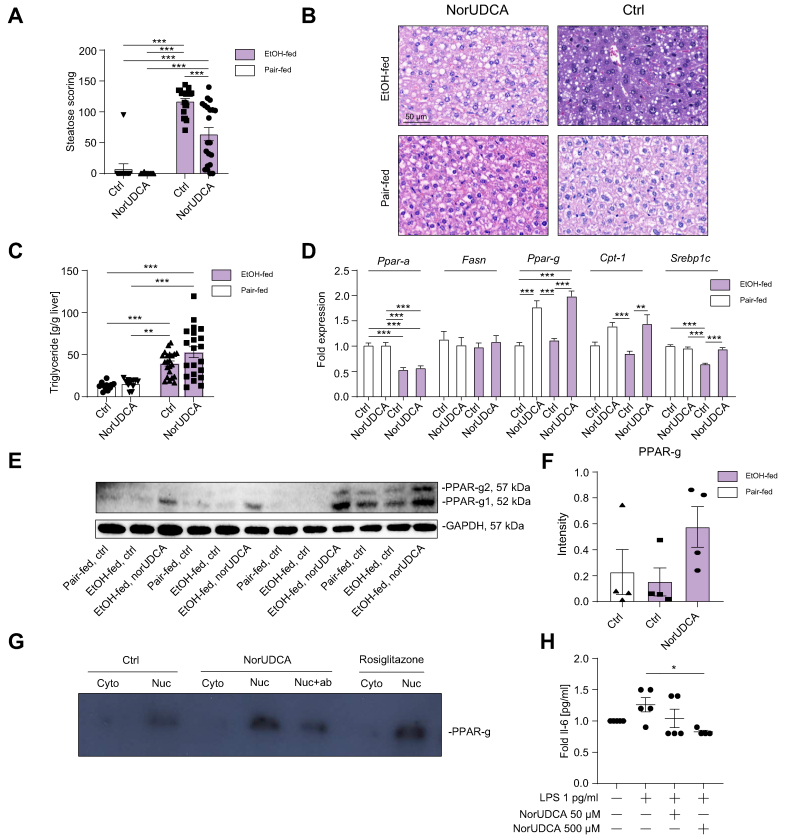
NorUDCA alters hepatic lipid metabolism by induction of PPARg. (A) NorUDCA treatment was associated with decreased hepatic steatosis scoring in EtOH-fed mice. (B) Representative H&E-stained liver sections. (C) EtOH feeding resulted in increased triglyceride accumulation, although no significant difference between EtOH-fed, norUDCA-treated mice and Ctrl could be observed. (D) NorUDCA treatment significantly increased the hepatic expression of *Pparg* in both EtOH-fed and pair-fed mice, and *Cpt-1* and *Srebp1c* in EtOH-fed mice. (E) Western blot analysis of PPARg1/2 and GAPDH and (F) quantification of PPARg1 and PPARg2. (G) Human primary immortalised hepatocytes were stimulated with norUDCA (500 μM), rosiglitazone (PPARg agonist, 10 μM), and Ctrl. PPARg was enhanced after norUDCA stimulation comparable with rosiglitazone in nuc. To confirm correct detection, PPARg antibody (nuc + ab) was used to decrease binding capability of the assay. (H) Human PBMCs were treated with LPS (1 pg/ml) and norUDCA (50 and 500 μM). IL-6 was measured after 24 h in the supernatant. NorUDCA decreased LPS-induced IL-6 response. Data are shown as mean ± SEM. ∗*p* <0.05, ∗∗*p* <0.01, and ∗∗∗*p* <0.001 according to one-way ANOVA with Bonferroni *post hoc* analysis or the Kruskal–Wallis test with Dunn’s *post hoc* analysis. β-actin was used as a housekeeping gene (D). Ctrl, control; *Cpt-1*, carnitine palmitoyltransferase 1; cyto, cytosol; EtOH, ethanol; *Fasn*, fatty acid synthetase; GAPDH, glyceraldehyde-3-phosphate dehydrogenase; LPS, lipopolysaccharide; norUDCA, 24-norursodeoxycholic acid; nuc, nuclear extracts; PBMC, peripheral blood mononuclear cell; *Pparg*, peroxisome proliferator-activated receptor gamma; *Srebp1c*, sterol regulatory element-binding protein 1c.

Hepatic steatosis was quantified on H&E-stained liver slides by an experienced board-certified liver pathologist based on a previously established scoring system.^37^ Ethanol feeding led to enhanced hepatic lipid accumulation (*p* <0.001; Fig. 3A and B), whereas norUDCA treatment significantly decreased the hepatic steatosis score in ethanol-fed mice (*p* <0.001; Fig. 3A and B). NorUDCA mainly tended to reduce microvesicular and mediovesicular hepatic steatosis in ethanol-fed mice (Fig. S3A and B), whereas macrovesicular steatosis was unchanged by norUDCA treatment compared with that in ethanol-fed controls (Fig. S3C). This could explain the tendency towards increased accumulation of triglycerides in the liver of ethanol-fed, norUDCA-treated mice compared with controls (Fig. 3C). In a next step, we performed quantitative PCR analysis of several genes involved in hepatic fatty acid metabolism. Interestingly, in ethanol-fed mice, norUDCA significantly increased the hepatic expression of *Pparg* (*p* <0.001; Fig. 3D), carnitine palmitoyltransferase 1 (*Cpt-1*) (*p* <0.01; Fig. 3D), and sterol regulatory element-binding protein 1 *(Srebp1c*) (*p* <0.001; Fig. 3D).

### NorUDCA shows potent anti-inflammatory properties via PPARg activation

PPARg (mainly isoform PPARg1) levels were increased upon norUDCA administration in ethanol-fed mice (Fig. 3E and F). As PPARg was shown to exhibit anti-inflammatory properties and to induce an anti-inflammatory (M2) macrophage phenotype, we assessed the activation of PPARg upon norUDCA stimulation. Using human immortalised hepatocytes and a luciferase assay, we could demonstrate an activation of PPARg by norUDCA (Fig. S3D). In a second step, immortalised human hepatocytes were stimulated with norUDCA (500 μM), rosiglitazone (PPARg agonist, 10 μM), and control for 48 h. Notably, PPARg was enhanced after norUDCA stimulation comparable with rosiglitazone in nuclear extracts (Fig. 3G). To further study the anti-inflammatory potential of norUDCA as indicated by a decreased expression of pro-inflammatory cytokines and an increased *Pparg* expression, we performed an *in vitro* experiment, stimulating peripheral blood mononuclear cells (PBMCs) of healthy donors with LPS and norUDCA (50 and 500 μM) for 24 h. IL-6 levels were significantly decreased in PBMCs stimulated with 500 μM norUDCA (*p* <0.05; Fig. 3H). Furthermore, we observed a trend towards increased *Pparg* expression in HepG2 cells upon norUDCA and LPS treatment (Fig. S3E).

### NorUDCA treatment alters intestinal microbiota composition

**Fig. 4:**
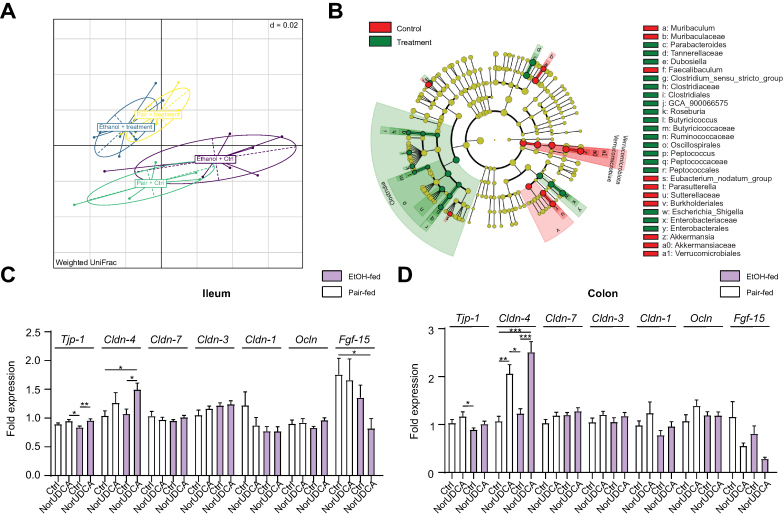
NorUDCA alters intestinal microbiota composition. (A) NorUDCA altered intestinal microbiota composition displayed by significant differences between groups in the principal coordinates analysis. (B) Analysis of relative abundances using LEfSe, indicating that multiple taxa are differentially relatively abundant in the caecal content of norUDCA-treated mice compared with Ctrl. (C) Ileal and (D) colonic expression of *Cldn-4* was increased by norUDCA in EtOH- and pair-fed mice. Data are shown as mean ± SEM. ∗*p* <0.05, ∗∗*p* <0.01, and ∗∗∗*p* <0.001 according to one-way ANOVA with Bonferroni *post hoc* analysis or the Kruskal–Wallis test with Dunn’s *post hoc* analysis. β-actin was used as a housekeeping gene (C and D). Ctrl, control; *Cldn*, claudin; EtOH, ethanol; *Fgf-15*, fibroblast growth factor-15; *Tjp-1*, tight junction protein 1; norUDCA, 24-norursodeoxycholic acid; Ocln, ocludin.

In a next step, we evaluated the influence of norUDCA on the intestinal microbiota composition. In weighted UniFrac principal coordinates analysis plots, a significant dissimilarity between all four investigated groups was observed (permutational multivariate ANOVA, *p* <0.001; Fig. 4A). At the genus level, in both norUDCA-treated mice groups, we observed a decrease of *Muribaculaceae-* and *Fecalibactulum-*related Amplicon Sequence Variants (ASVs), compared with that in controls. *Roseburia*-, *Enterobacteriaceae*-, and *Clostridium*-related ASVs, by contrast, were relatively more abundant in norUDCA-treated mice (Fig. 4B). Moreover, norUDCA supplementation upregulated the expression of ileal claudin 4 (*Cldn-4*) (*p* <0.05; Fig. 4C) and tight junction protein 1 (*Tjp-1*) (*p* <0.01; Fig. 4C) in ethanol-fed mice. In the colon, norUDCA treatment significantly increased the expression of *Cldn-4* in pair-fed mice (*p* <0.01; Fig. 4D) as well as in ethanol-fed mice (*p* <0.001; Fig. 4D), which might reflect improved gut integrity. Ethanol-induced increase of circulating LPS tended to be lower in ethanol-fed, norUDCA-treated mice than in controls (Fig. S3F). LPS-binding protein (*LBP*) expression was also not different within the groups (Fig. S3G).

### Therapeutic norUDCA treatment ameliorates experimental ALD

**Fig. 5:**
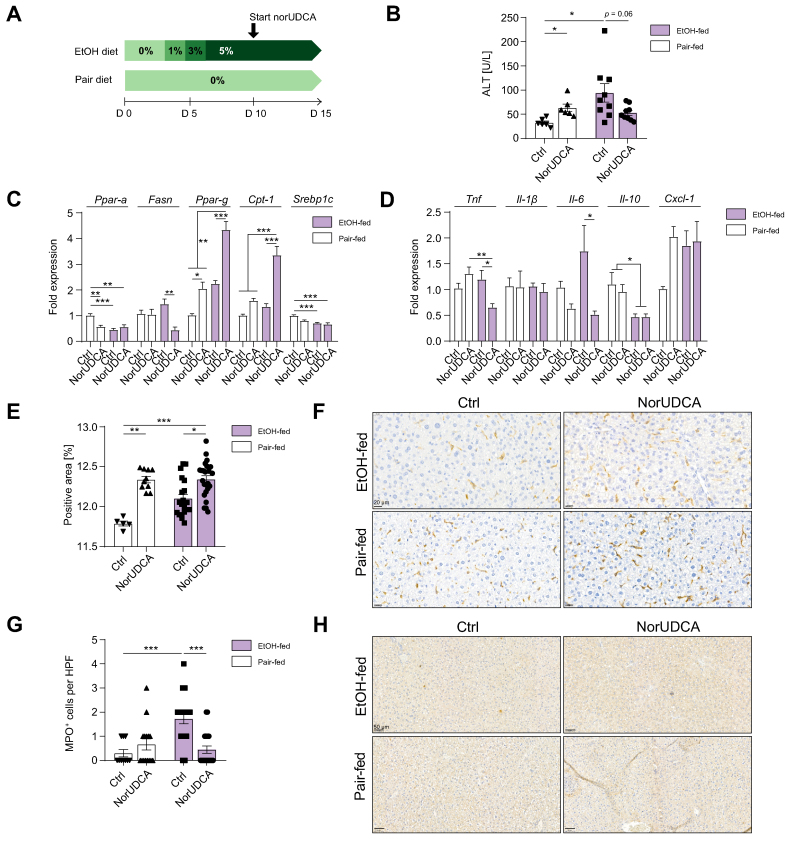
Therapeutic norUDCA supplementation ameliorates experimental ALD. (A) Graphical illustration of experimental design. (B) ALT concentration tended to be decreased upon norUDCA in EtOH-fed mice. (C) Hepatic expression of *Fasn*, *Pparg,* and *Cpt-1* was altered by norUDCA. (D) Hepatic expression of pro-inflammatory cytokines including *Tnf*, *Il-6*, and *Il-10* was decreased by norUDCA in EtOH-fed mice. (E) Quantification of F4/80^+^-positive area per HPF based on F4/80 immunoreactivity. (F) Representative images of F4/80^+^-stained liver sections (brown indicates F4/80^+^ cells). (G) Number of MPO^+^ cells was decreased in norUDCA-treated, EtOH-fed mice compared with Ctrl. (H) Representative images of MPO-stained liver sections (brown indicates MPO^+^ cells). Data are shown as mean ± SEM. ∗*p* <0.05, ∗∗*p* <0.01, and ∗∗∗*p* <0.001 according to one-way ANOVA with Bonferroni *post hoc* analysis or the Kruskal–Wallis test with Dunn’s *post hoc* analysis. β-actin was used as a housekeeping gene (C and D). ALT, alanine aminotransferase; *Cpt-1*, carnitine palmitoyltransferase 1; ALD, alcohol-related liver disease; Ctrl, control; *Cxcl-1*, C-X-C motif chemokine ligand 1; EtOH, ethanol; *Fasn*, fatty acid synthetase; HPF, high-power field; MPO, myeloperoxidase; norUDCA, 24-norursodeoxycholic acid; *Ppar*, peroxisome proliferator-activated receptor; *Srebp1c*, sterol regulatory element-binding protein 1c; *Tnf*, tumour necrosis factor.

To further assess the potential therapeutic effect of norUDCA in already established experimental ALD, mice were fed a Lieber–DeCarli diet for 15 days, and norUDCA supplementation was started at Day 10 (Fig. 5A). Ethanol feeding resulted in significantly increased ALT levels compared with that in pair-fed control mice, whereas norUDCA treatment decreased the ALT by 45% in ethanol-fed mice (*p* = 0.06; Fig. 5B). Serum ethanol concentration was not significantly different between the groups (Fig. S4A). Gallbladder weight, as a surrogate marker for increased bile flow, was significantly higher in norUDCA-treated mice in both the ethanol- and pair-fed groups (Fig. S4B). Eventually, the hepatic expression of *Mrp4* was increased in ethanol-fed mice, but not pair-fed mice, upon norUDCA administration (Fig. S4C). NorUDCA increased the hepatic expression of *Pparg* (*p* <0.001; Fig. 5C) and *Cpt-1* (*p* <0.001; Fig. 5C) in both pair-fed and ethanol-fed mice, whereas norUDCA decreased fatty acid synthase (*Fasn*) expression only in ethanol-fed mice (*p* <0.01; Fig. 5C). On the protein level, CPT-1 was augmented in ethanol-fed, norUDCA-treated mice (Fig. S4D and E).

NorUDCA induced a reduction of hepatic *Tnf* (*p* <0.05) and *Il-6* (*p* <0.05) expression in ethanol-fed mice (Fig. 5D). Similar to that in the preventive setting, norUDCA treatment increased the number of F4/80^+^ cells in ethanol- and pair-fed mice (Fig. 5E and F). Myeloperoxidase-positive neutrophils were increased by ethanol feeding compared with those in controls but were lowered upon norUDCA treatment in ethanol-fed mice (*p* <0.001; Fig. 5G and H).

## Discussion

We investigated the efficacy of norUDCA in experimental ALD. Although ALD is one of the most frequent liver diseases, with alcohol-related liver cirrhosis being associated with 22.2 million disability adjusted life years in 2016, therapeutic options are still limited.^38^ Different mechanisms contribute to the development of ALD, including ethanol toxicity, gut barrier dysfunction, and endotoxaemia.^5^^,^^6^^,^^8^^,^^39^ Notably, bile acids may also play an important role in the pathogenesis of ALD.^9^

In this study, we demonstrated that the administration of norUDCA protected wild-type mice from ethanol-induced liver injury in a preventive and therapeutic setting. Furthermore, we unravelled several potential mechanisms how norUDCA, a side chain shortened homologue of UDCA, ameliorates experimental ALD. Specifically, we demonstrated that norUDCA treatment ameliorated serum concentrations of ALT, reduced hepatic cell death, and decreased hepatic expression of pro-inflammatory cytokines, such as *Tnf*, *Il-6*, and *Il-1β.* The amelioration of pro-inflammatory pathways could potentially be explained by an increased expression of anti-inflammatory *Pparg* in norUDCA-treated mice. In addition, norUDCA could alter the intestinal microbiota in both ethanol- and pair-fed mice.

NorUDCA, was primarily tested in the treatment of cholestatic liver diseases such as PSC. In an experimental PSC model, *Mdr2*^*-/-*^ mice treated with norUDCA showed reduced hepatic inflammation.^25^ Furthermore, norUDCA was proven to affect cells of the innate and adaptive immune system such as macrophages and CD8^+^ T cells in models of experimental cholestatic liver diseases.^30^ In hepatocyte-specific NF-κB essential modulator (NEMO)-deficient mice, a genetic model for NAFLD, norUDCA attenuated liver damage, depicted by decreased transaminases, histological improvement, and reduced hepatic fibrosis.^27^ The anti-inflammatory properties of norUDCA were further observed in a model of *Schistosoma mansoni*-induced liver injury, whereas norUDCA treatment attenuated liver inflammation by reducing the expression of MHC class II molecules on antigen-presenting cells including macrophages.^28^

Likewise, in ALD, macrophages represent a cornerstone in the pathogenesis of the disease.^40^ Under numerous stimulating factors in their microenvironment, macrophages may polarise into either pro-inflammatory M1 or anti-inflammatory M2 phenotype. Whereas the macrophage phenotype M1 is induced by LPS and interferon gamma, IL-13 and IL-4 activate M2 macrophages.^41^ Ethanol administration likewise induces the polarisation of M1 macrophages through NF-κB signalling.^20^^,^^21^^,^^42^

In our study, we found increased numbers of F4/80^+^ macrophages after norUDCA administration in ethanol-fed mice as well as in pair-fed mice. Interestingly, macrophages showed a pronounced M2 phenotype depicted by an increased *Arg*/*iNos* expression only in norUDCA-treated, ethanol-fed mice. We were further able to demonstrate the anti-inflammatory properties of norUDCA on macrophages *in vitro*. NorUDCA treatment attenuated pro-inflammatory cytokine production upon LPS stimulation of PBMCs. In conclusion, we could show an amelioration of the ethanol-induced liver injury by norUDCA, possibly mediated by enhanced macrophage M2 polarisation.

PPARg is a nuclear receptor with potent effects in metabolic and inflammatory pathways that may be present in different isoforms,^43^ namely, PPARg1 and PPARg2. Whereas PPARg1 shows anti-inflammatory properties and is expressed in macrophages,^44^^,^^45^ PPARg2 is mainly involved in lipid storage and is found in adipocytes as well as in hepatocytes.^46^ In the course of exploring new therapeutic agents for fatty liver disease, PPARg agonists (such as rosiglitazone) were tested. Different studies found improved metabolic parameters and antisteatogenic effects in NAFLD upon rosiglitazone treatment, serving as a potent inducer of PPARg.[47], [48], [49], [50] Although chronic ethanol consumption was associated with an activation of PPARg2,^51^ PPARg2^-/-^ mice had decreased ethanol-induced liver injury.^52^ Nevertheless, the role of PPARg1 in ALD is currently unclear.

Notably, in our study, we found a significantly enhanced expression of *Pparg* upon norUDCA administration, both in the preventive as well as therapeutic setting and in pair-fed as well as ethanol-fed mice. Moreover, we could demonstrate a strong induction of anti-inflammatory PPARg1 in livers of norUDCA-treated, ethanol-fed mice. The induction of PPARg by norUDCA was further confirmed by two different *in vitro* assays. Interestingly, glitazones were tested in a clinical trial to treat alcohol addiction,^53^ but the trial was stopped early because of increased craving in pioglitazone-treated patients as a result of augmented neuroendocrine stress response to LPS. In line with these data, PPARg agonists were not influencing IL-6 and TNF levels in macrophages *in vitro* or *in vivo*.^54^ Therefore, an alternative strategy to target PPARg without facing the adverse effects of its synthetic ligands could be norUDCA.

Similarly, Beraza *et al.*^27^ observed tendency towards increased *Pparg* expression after UDCA administration compared with that in controls, whereas norUDCA decreased the expression in *NEMO*^*-/-*^ mice. Interestingly, PPARg is an important regulator of both cell differentiation and polarisation of macrophages induced by NF-κB and IL-4/IL-13,^20^^,^^22^^,^^55^ which also might explain the increase of anti-inflammatory M2 macrophage polarisation in our study. Wagner *et al.*^56^ found improved endothelial barrier and reduced inflammatory parameters in mice treated with ethanol, LPS and rosiglitazone compared to ethanol- and LPS-treated controls. In conclusion, norUDCA induces PPARg activation, and this effect might contribute to its anti-inflammatory mode of action in the liver, mainly by ameliorating the hepatic cytokine response and induction of an M2 macrophage phenotype.

Changes within the intestinal microbiota composition and alcohol-induced liver disease are closely associated. Alcohol overconsumption results in microbial changes, but different bacterial strains may influence disease progression. In weighted UniFrac principal coordinates analysis plots, all four observed groups were significantly different, suggesting that not only ethanol feeding but also norUDCA treatment changed the intestinal microbiota composition in our experiments. Comparing norUDCA treatment groups with controls, we could observe a decreased abundance of *Muribaculaceae-* and *Fecalibactulum-*related ASVs. *Muribaculaceae,* a not well-described strain, was increased by UDCA treatment in high-fat-diet mice.^57^ *Faecalibacteria* are known as beneficial butyrate producers in the human gut. A recent study found increased *Faecalibacterium prausnitzii* in patients treated with UDCA.^58^ Llopis *et al.*^59^ found decreased *Faecalibacterium* abundance in patients with ALD. In our study, we observed an increased abundance of *Roseburia-*related ASVs. The administration of *Roseburia* in a model of ALD resulted in the improvement of hepatic steatosis and inflammation,^60^ suggesting a beneficial role of this strain in ALD.

In summary, we could demonstrate efficacy of norUDCA in experimental ALD, which might be caused by an increase in hepatic PPARg1 and enhanced macrophage polarisation towards an anti-inflammatory M2 phenotype. Future clinical trials for norUDCA in ALD are now warranted to prove its efficacy in ALDs in humans.

## Financial support

HT is supported by the excellence initiative VASCage (Centre for Promoting Vascular Health in the Ageing Community), an R&D K-Centre (COMET program – Competence Centers for Excellent Technologies) funded by the 10.13039/501100004956Austrian Ministry for Transport, Innovation and Technology, the Austrian Ministry for Digital and Economic Affairs, and the federal states Tyrol, Salzburg, and Vienna. CG is supported by the Austrian Society of Gastroenterology and Hepatology (ÖGGH) and received speaker fees from Abbvie. TEA is supported by the Austrian Science Fund (FWF P33070) and by the 10.13039/501100000780European Union *(*ERC-STG Grant agreement No. 101039320). JS is supported by the Austrian Society of Gastroenterology and Hepatology (ÖGGH) and the German Society of Inflammatory Bowel Disease (DACED). MT is supported by the Austrian Science Fund (FWF) F7310. He received speaker fees from Falk Foundation, Gilead, Intercept, and MSD; he advised for Abbvie, Albireo, BiomX, Boehringer Ingelheim, Falk Pharma GmbH, Genfit, Gilead, Hightide, Intercept, Janssen, MSD, Novartis, Phenex, Pliant, Regulus, Siemens, and Shire. He further received travel grants from Abbvie, Falk, Gilead, and Intercept and research grants from Albireo, Alnylam, Cymabay, Falk, Gilead, Intercept, MSD, Takeda, and UltraGenyx. He is also the co-inventor of patents on the medical use of norUDCA filed by the Medical Universities of Graz and Vienna.

## Authors’ contributions

Designed, performed, and analysed the animal and *in vitro* experiments together: CG, MM, SD, TC. Performed histologic analysis: GO. Performed microbiota analysis: BH, PP. Provided expertise and edited the manuscript: TEA, FG, JS. Prepared the manuscript: CG, MM. Coordinated the project: MT, HT.

## Data availability statement

The data that support the findings of this study are available from the corresponding author upon reasonable request.

## Conflicts of interest

The authors declare no conflict of interest.

Please refer to the accompanying ICMJE disclosure forms for further details.
