# Supplementary material for: 24-Norursodeoxycholic acid ameliorates experimental alcohol-related liver disease and activates hepatic PPARγ
Source: JHEP Rep. 2023 Aug 3;5(11):100872. doi: 10.1016/j.jhepr.2023.100872 (PMC10561126; doi:10.1016/j.jhepr.2023.100872)
Supplement: Multimedia component 1 [file mmc1.pdf]

24-Norursodeoxycholic acid ameliorates experimental alcohol-related liver disease and activates hepatic PPARγ

C. Grander, M. Meyer D. Steinacher, T. Claudel, B. Hausmann, P. Pjevac, F. Grabherr, G. Oberhuber, M. Grander, N. Brigo, A. Jukic, J. Schwärzler, G. Weiss, T.E. Adolph, M. Trauner, H. Tilg

Table of contents

Supplementary materials and methods 2

Supplementary figures.. 9

Supplementary references... 15

Supplementary materials and methods

Immunohistochemistry and TUNEL labelling

Liver sections were deparaffinised in xylene and dehydrated in an ethanol gradient. Antigen unmasking was performed by using a 2 % citrate-buffer (pH = 6, Vector Laboratories, Burlingame, USA) in a conventional steamer. Inactivation of endogenous peroxidase activity was carried out by using Peroxidase Blocking Solution (Dako, Santa Clara, CA, USA), protein blocking was performed with a ready-to-use kit (MP-740; Dako, Santa Clara, CA, USA). Afterwards, antibodies (MPO, Dako, Santa Clara, CA, USA; F4/80, Cell Signaling Technology #70076) and secondary anti-rabbit antibodies (Dako, Santa Clara, CA, USA) were applied. To visualize immunoreactivity, ImmPACT AMEC (Vector Laboratories, Burlingame, CA, USA) was used, followed by staining with DAB (Dako, Santa Clara, CA, USA) for 2 minutes and counterstaining with hematoxylin (Dako, Santa Clara, CA, USA) for 20 seconds. MPO^+ and $F4/80^+$ cells were counted in 10 randomly selected fields of $1mm^2$. TUNEL labeling was performed according to the manufacturer's instructions (Roche, 11684817910). $TUNEL^+$ cells were quantified in 10 randomly selected fields of $1mm^2$ by counting TUNEL+ cells per HPF.

Triglyceride measurement

Frozen liver tissue samples were homogenized in PBS (volume was adjusted to the liver tissue weight) followed by an incubation at 60° C for 30 minutes. Afterwards, samples were centrifuged (12.000 G, 10 min, room temperature) and supernatant was taken off. Triglyceride concentration was measured with TG-Reagent (Roche, Basel, Switzerland) according to the manufacturer instructions.

Ethanol measurement

Ethanol concentration in serum samples was measured using the EnzyChrom assay (ECET-100, BioAssay Systems, Hayward, CA, USA) according to the manufacturer instructions.

Western blot

Hepatic protein was isolated using T-PER tissue protein extraction reagent. The reagent was supplemented with HALT proteinase inhibitor cocktail (Thermo Fisher Scientific, Waltham, MA, USA). Protein concentrations were measured by Bradford Protein Assay (BioRad, 5000006), separated by SDS-PAGE (Hercules, Bio Rad, CA, USA) and blotted onto Hybond-P PVDF membranes (GE Healthcare, Chicago, IL, USA). Blocking of membranes was performed with 5% skim milk. Membranes were incubated overnight with the primary antibody. Following antibodies were used for detection: PPARg, Cell Signaling Technology #2443; PPARg A3409A, Invitrogen; CPT1A, Cell Signaling Technology #97361; GAPDH, Cell Signaling Technology #2118. Visualization of immunoreactivity was carried out by using HRP-conjugated secondary antibodies (Cell Signalling Technology, #7074) and ECL Select Western Blotting Detection Reagent (Amersham, RPN2235). GAPDH (GAPDH, glyceraldehyde 3-phosphate dehydrogenase) was used as a reference protein. Quantitation of the western blot signals was performed using the Biorad ChemiDoc MP (Hercules, CA, USA). Densitometry of immunoblots was performed with BioRad Image Lab software.

FACS Analysis:

Bone marrow derived macrophages (BMDM) from C57BL/6N mice were prepared as described (Brigo et al. 10.21769/BioProtoc.4440). After 5 days of differentiation with 50 ng/ml macrophage colony stimulating factor (MCSF, Peprotech), cells were subjected to lipopolysaccharide (LPS) (100 ng/mL; Invivogen, San Diego, CA, USA), Interferon-γ for M1 polarization (100 ng/mL; Peprotech, London, United Kingdom) or Interleukin 4 for M2 polarization (10ng/ml; Peprotech, London, United Kingdom) or norUCDA stimulation for 24h. The following day, cells were scraped and stained for FACS analysis as previously described [1]. Percentage of M1 (iNOS+; CD80+) or M2 (Arg1+; CD206+) macrophages was determined by flow cytometry analysis using a CytoFLEX S (Beckman Coulter). The gating strategy is shown in Fig. S2. Bars indicates mean +- SEM.

ELISA:

IL-6 concentration was measured using commercially available ELISA kits from R&D systems (Minneapolis, MN, USA) according to the manufacturer's Instructions.

Microbiome-Studies:

The obtained sequence data was quality-filtered and demultiplexed, followed by amplicon sequencing variant (ASV) inference with DADA2 [2](4), enabling analysis at the highest possible taxonomic resolution. Resulting ASV sequences were taxonomically classified using SINA [3] with the newest release of the the SILVA SSU rRNA database [4]. If necessary, contaminants were removed in silico using the decontam software package [5].

Abundance measurements (counts) of ASVs, as well as ASV sum counts at higher taxonomic levels were statistically evaluated, to test for significant differences in microbial community composition between the subject groups. Detection of significantly more abundant amplicon sequence variants in the case over the controls weas performed, and adjusted P-values were calculated using the Benjamini-Hochberg method and differences supported with P-values < 0.05 were considered significant.

Statistical analysis was performed with the metagenomeSeq software, which has been proven optimal for amplicon datasets [6]. MetagenomeSeq normalized the abundance data to address varying depths of sequencing coverage across samples, and then a zero-inflated log-normal mixture model was applied to calculated the fold changes between the case and control group for each taxonomic level [7].

<u>In-vitro studies:</u>

Inflammation analysis: Peripheral Blood Mononuclear Cells (PBMCs) were collected from five healthy volunteers. Blood samples were collected in heparinized tubes, followed by density gradient centrifugation of whole blood samples on Lymphoprep solution according to the manufacturer's instructions (Axis Shields, Oslo, Norway) [8]. After isolation, PBMCs were stimulated with lipopolysaccharide (LPS) (1 pg/mL; Invivogen, San Diego, CA, USA) for 24 hours and 50µM as well as 500µM norUDCA. DMSO served as negative control. 24 hours after stimulation, cells supernatant was harvested and IL-6 was measured by ELISA.

Cell culture and luciferase assay:

Human primary immortalized hepatocytes (IHH) [9] were adapted to DMEM with 10% fetal bovine serum (FBS) and 1% penicillin/streptomycin (all from Thermo Fischer, Vienna, Austria). IHH were seeded in a 24 well-plate, then transiently transfected with 150 ng/well of PPARg response element (located into the promoter of PPARg [10] cloned in 3 copies in front of a minimum thymidine kinase promoter linked to a luciferase (TK-luc-pGl3) construct and 100ng of pSG5-PPARg2 expressing plasmid (a gift from Dr Johan Auwerx, EPFL Lausanne, Switzerland) using Fugene transfection reagent (Promega, Madison, WI) in sterile DMEM without FBS for 12 h. Medium containing 10% FBS was then added for 24 h with NorUDCA 500 µM and the cells lysed using a lysis solution (4% Triton-X100, Glycyl-Glycine 100mM, $MgSO_4$ 100mM, EGTA 250mM) for 1 h at room temperature on a shaker. Cell extracts were then combined with the luciferin solution (Luciferin 2.5mM and ATP 20mM, Merck, Vienna) and analyzed with a luminometer (Lumat LB9507 EG&G Berthold, Germany).

Electrophoretic mobility shift assay (EMSA):

IHH cells were cultivated for 48 h with or without NorUDCA 500µM or rosiglitazone 10µM (Merck, Vienna, Austria) in DMEM without fetal calf serum, with 0.5 % penicillin/streptomycin (ThermoFisher Scientific, Vienna, Austria) and nuclear extracts (NE) were prepared. Briefly, cells were centrifuged for 5 min at 1,000 rpm, the pellet dissolved in homogenization buffer (15mM Tris HCl, pH 8; 15mM NaCl, 60mM KCl; 0.5mM EDTA; 1mM PMSF and 1mM beta mercaptoethanol). After centrifugation during 5 min at 1,000 rpm, cell pellets were suspended in Hypotonic Buffer (HB) containing 0.05 % Triton X-100. After another centrifugation at 1,200 rpm the supernatant (cytosolic fractions) were collected and kept at -80°C. The pellets were

washed with 5 ml of HB buffer with Triton and centrifuged for 10 min at 1,200 rpm, the pellet was washed with 5 ml of HB buffer without Triton and centrifuged at 1,200 rpm for 10 min. Finally, pellets were suspended in 50 µl of HB buffer modified with 360mM KCl and incubated at 4°C for 30 min, before a final centrifugation for 5 min at 13,000 rpm. The supernatants (nuclear fractions) were kept at -80°C before use in gel shift. Protein concentrations were measured by the bicinchoninic assay (ThermoFisher, Vienna, Austria). Double-stranded oligonucleotides (sequence 5′-GATCCTAGAATATAGGTCAGGGAAG-3) were end labeled with γ-32P ATP (Hartmann Analytic, Braunschweig, Germany) using T4- polynucleotide kinase (New England Biolabs, Frankfurt am Main, Germany) and purified by column elution using the QIAquick nucleotide removal kit (Qiagen, Hilden, Germany). Oligonucleotides and cytosolic and nuclear extracts (1 µg) were mixed in a DNA binding buffer containing in a 20µl final volume (10mM Tris pH 7.5, 150mM NaCl, 1 mMDTT, 1mM EDTA, 5% glycerol (Sigma-Aldrich, Vienna, Austria), for 10 minutes at room temperature before the radiolabeled probe (0.5 ng) was added. Binding reactions were further incubated for 10 minutes with PPARg antibody (sc-7273X, Santa-Cruz biotechnology, Heidelberg, Germany) and resolved by 4 % non-denaturing polyacrylamide gel electrophoresis in 0.25X Tris-Borate-EDTA (TBE) buffer at room temperature. After run, gel was dried for 1h at 60°C and transferred into developing cassette (Biomax, Kodak) for overnight film exposure at -80C.

Quantification of alanine aminotransferase (ALT) in mouse serum

The quantification of murine ALT (alanine aminotransferase) in serum samples was performed by using an enzymatic assay (BQ-Kit, San Diego, CA, USA) according to the manufacturer instructions.

Culture and stimulation of HepG2

HepG2s were cultured in RPMI (supplemented with 10% fetal calf serum, Sigma, St. Louis, MO, USA) as described above [11]. Cells were stimulated with recombinant human IL-6 (10 ng/ml; Peprotech Austria, Vienna, Austria), recombinant IL-1β (1 ng/ml, Peprotech Austria, Vienna, Austria) or lipopolysaccharide (LPS) (100 ng/mL; Invivogen, San Diego, CA, USA) for four and 24 hours. As negative control cells were stimulated with DMSO. Four and 24 hours after stimulation, cells were harvested and RNA was extracted and further processed as described above.

RNA isolation and PCR of liver tissue

Tissue samples were homogenized in TRIzol (Thermo Fisher Scientific, Waltham, MA, USA) using a metal bead homogenizing system (Precellys, Bertin Technologies, Montigny-le-Bretonneux, France) followed by RNA isolation. Reverse transcription was accomplished with Reverse Transcription System (Thermo Fisher Scientific, Waltham, MA, USA), followed by quantitative real-time PCR using SybrGreen (Eurogentec, Seraing, Belgium) and the Mx3000 qPCR cycler (Stratagene California, San Diego, CA, USA). βActin was used as a reference gene. All used PCR primers are available on request.

Supplementary figures

Supplementary Figure 1

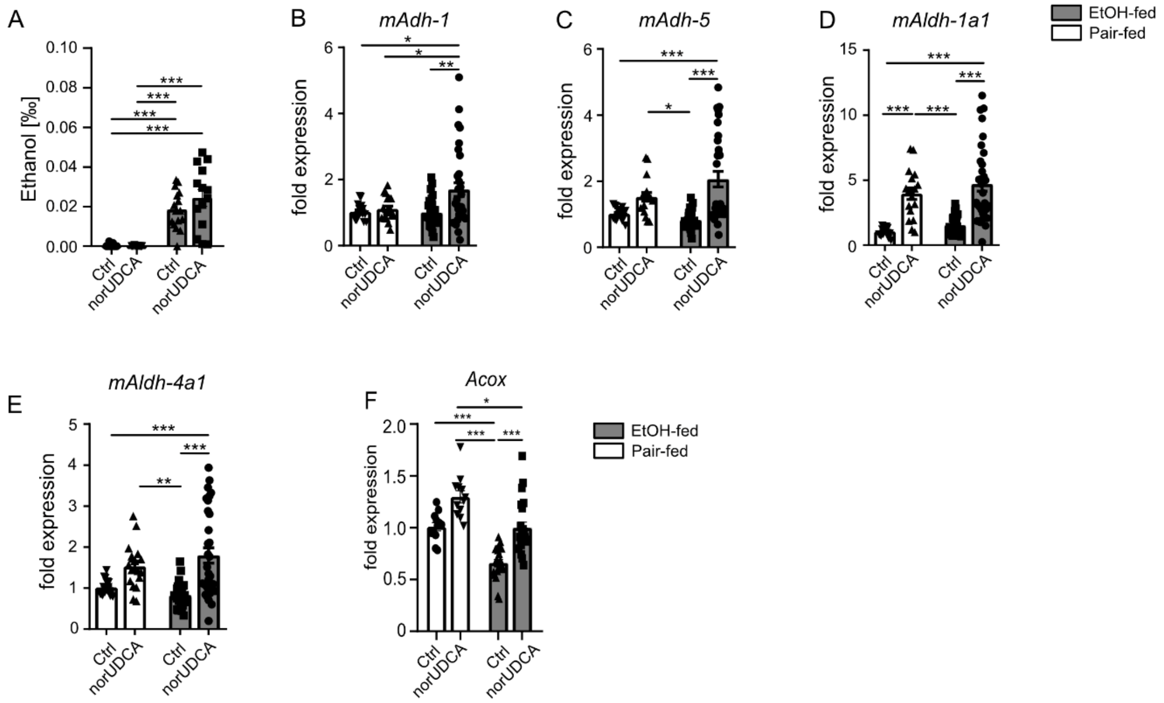

Fig. S1: (A) Shown is the serum concentration of ethanol. Data are shown as mean ± SEM. *p<0.05; **p<0.01; ***p<0.001 according to one-way ANOVA with Bonferroni post-hoc analysis. Acox, peroxisomal acyl-coenzyme A oxidase 1; Adh, alcohol-dehydrogenase; aldh, aldehyde- dehydrogenase; Ctrl, control; EtOH, ethanol.

Supplementary Figure 2

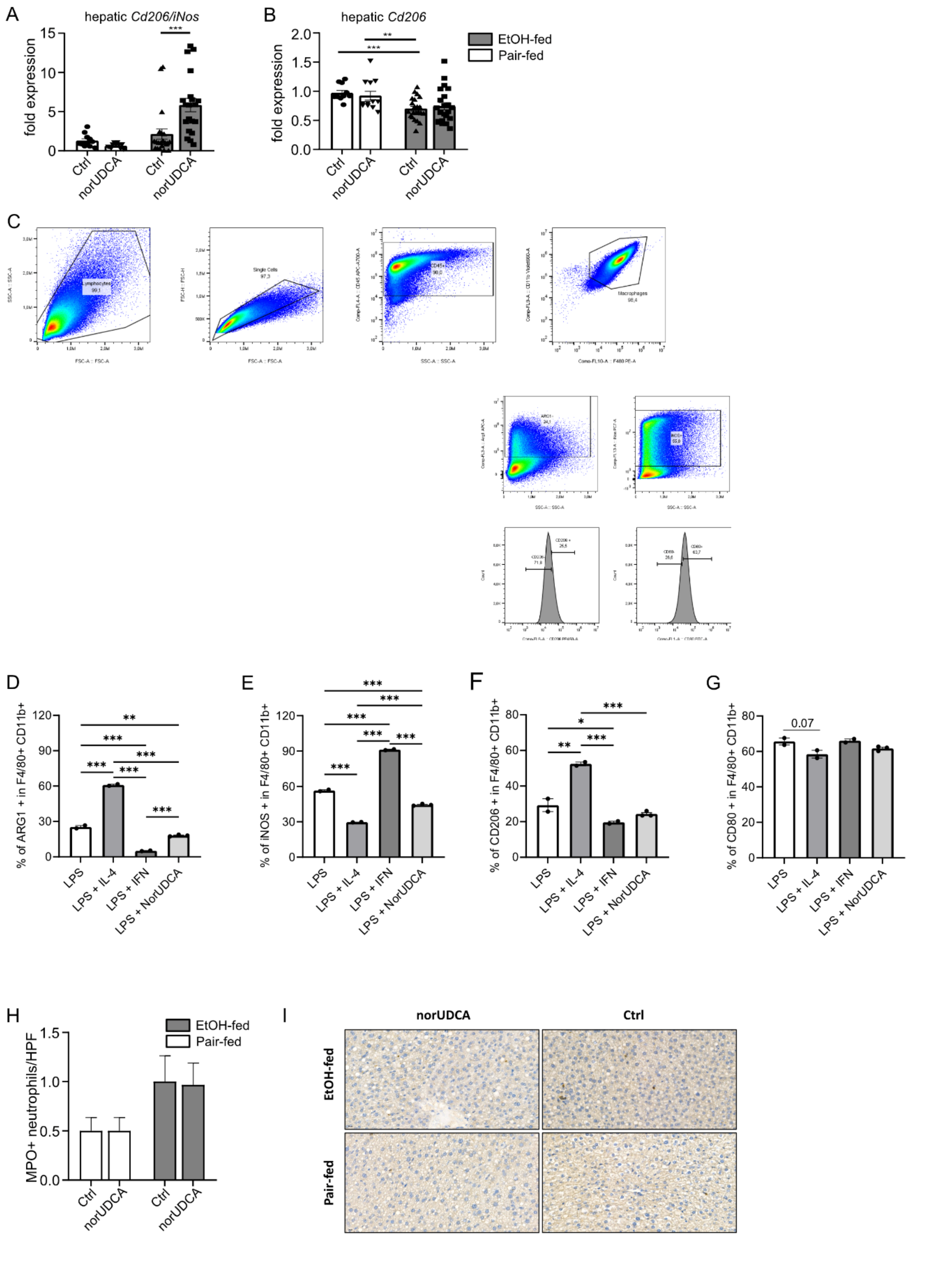

Fig. S2: (A) Shown is the hepatic mRNA expression ratio of *Cd206/iNos* (B) and *Cd206*. (C) Bone marrow derived macrophages were stimulated with 100 ng/ml LPS;

100 ng/ml IFNγ; 10 ng/ml IL-4 and 500µM norUDCA. After 24h of stimulation BMDM were scraped, stained and analyzed with FACS. Gating strategy for single cells, CD45+, CD11b+ F4/80+ double positive cells. To differentiate between M1 and M2 macrophages, iNOS+ and CD80+ (M1) cells as well as ARG1+ and CD206+ (M2) cells were determined. Quantification of ARG1+ (D), iNOS+ (E), CD206+ (F), CD80+ (G) BMDMs, determined by flow cytometry and presented in percent of F4/80+CD11b+ single cells indicating macrophage polarization towards M1 (E, G) and M2 (D, F) phenotype. (H) Number of MPO^+ cells was unchanged after norUDCA treatment in ethanol-fed mice compared to controls. (I) Representative images of MPO stained liver sections (brown indicates MPO^+ cells, black arrow). Data are shown as mean ± SEM. *$p<0.05$; **$p<0.01$; ***$p<0.001$ according to one-way ANOVA with Bonferroni post-hoc analysis or Kruskal-Wallis test with Dunn's post-hoc analysis. β-Actin was used as a house-keeping gene (A-B). Ctrl, control; EtOH, ethanol; LPS, lipopolysaccharide; iNOS, Inducible nitric oxide synthase; IL, interleukin; IFN, interferon; MPO, myeloperoxidase.

Supplementary Figure 3

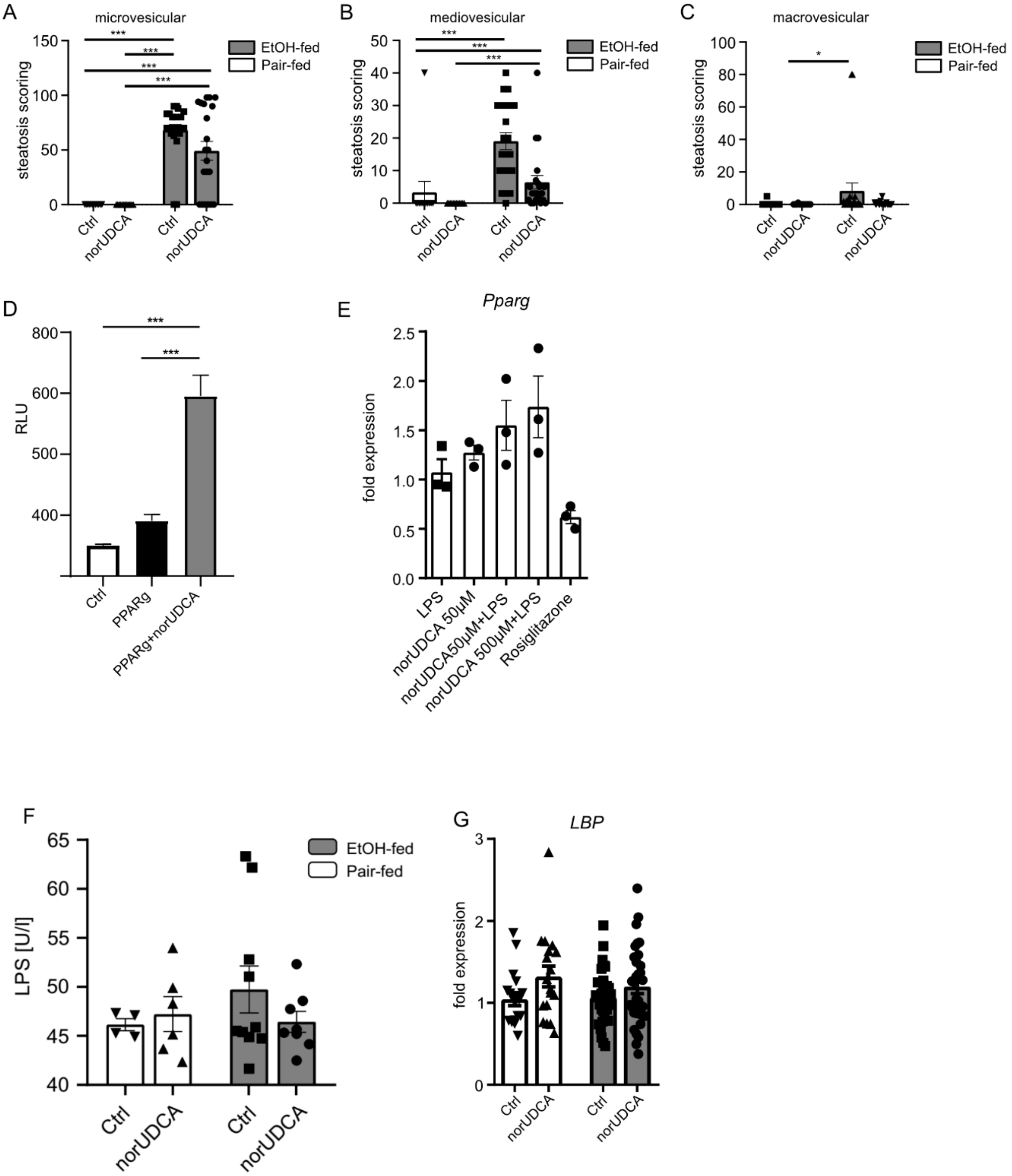

Fig. S3:

Staging of hepatic steatosis based on (A) microvesicular (B) mediovesicular (C) macrovesicular steatosis. (D) Luciferase assay with human primary immortalized hepatocytes was used to test PPAR-gamma activation upon norUDCA stimulation. (E)

Pparg mRNA expression after treatment of HepG2 cells with norUDCA and LPS compared to house-keeping gene ß-actin. (F) Serum LPS concentration of ethanol- and Pair-fed mice with and without norUDCA treatment. (G) LPS-binding protein (LBP) mRNA expression is not significantly altered between the groups. Data are shown as mean ± SEM. *p<0.05; **p<0.01; ***p<0.001 according to one-way ANOVA with Bonferroni post-hoc analysis or Kruskal-Wallis test with Dunn's post-hoc analysis. β-Actin was used as a house-keeping gene (E, G). Ctrl, control; EtOH, ethanol; LBP, LPS binding protein; LPS, lipopolysaccharide; Pparg, peroxisome proliferator-activated receptor gamma.

Suppl Figure 4

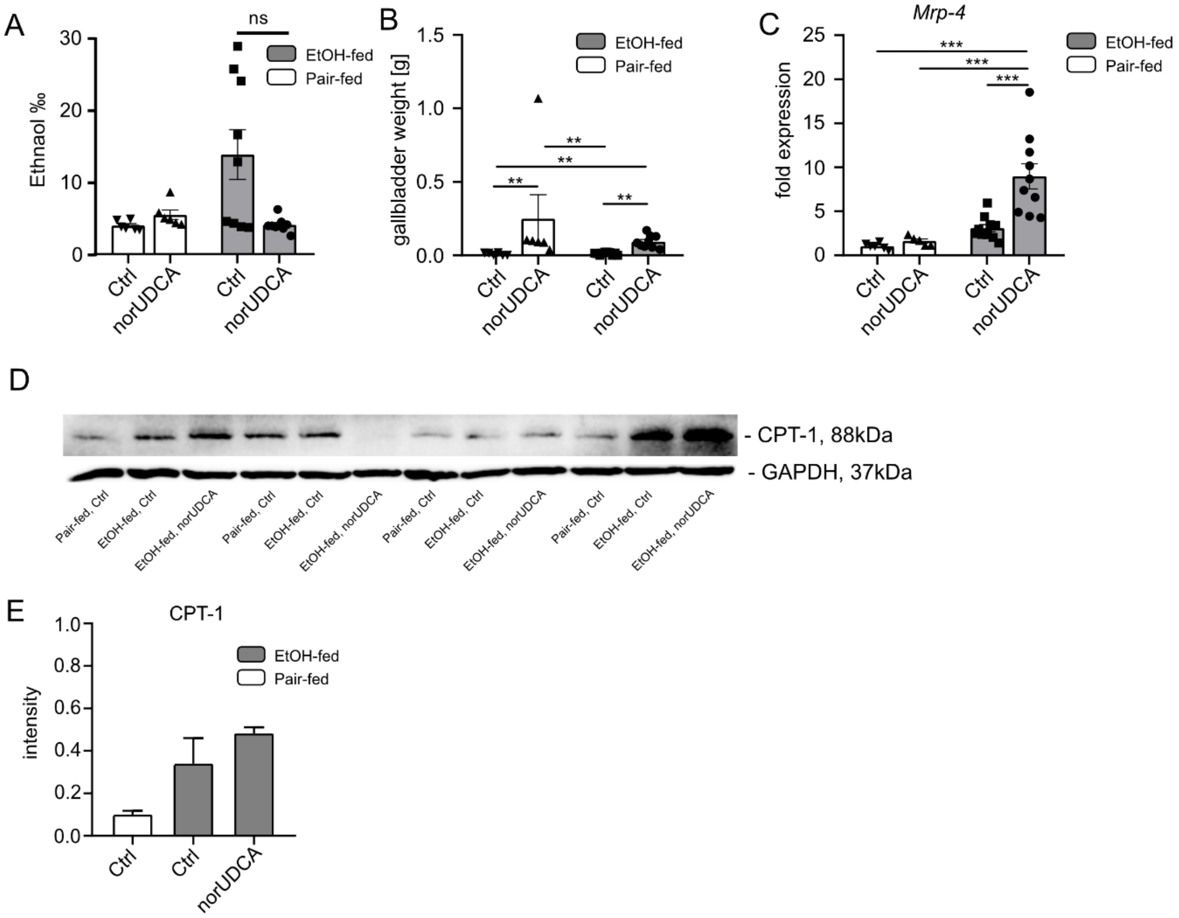

Fig. S4:

(A) Shown is the serum concentration of ethanol. (B) Gallbladder weight was increased upon norUDCA treatment. (C) Hepatic expression of *Mrp-4.* (D) Westernblot analysis of CPT-1 with GAPDH with (E) quantification of CPT-1. Data are shown as mean ± SEM. *p<0.05; **p<0.01; ***p<0.001 according to one-way ANOVA with Bonferroni post-hoc analysis or Kruskal-Wallis test with Dunn's post-hoc analysis. β-Actin was used as a house-keeping gene (C). Ctrl, control; EtOH, ethanol, Cpt-1, Carnitine palmitoyltransferase 1; GAPDH, Glyceraldehyde-3-phosphate dehydrogenase, MRP-4, multidrug resistance-associated protein 4.

Supplementary references

[1] Brigo N, Pfeifhofer-Obermair C, Tymoszuk P, Demetz E, Engl S, Barros-Pinkelnig M, et al. Cytokine-Mediated Regulation of ARG1 in Macrophages and Its Impact on the Control of Salmonella enterica Serovar Typhimurium Infection. Cells 2021;10.

[2] Callahan BJ, McMurdie PJ, Rosen MJ, Han AW, Johnson AJ, Holmes SP. DADA2: High-resolution sample inference from Illumina amplicon data. Nature methods 2016;13:581-583.

[3] Pruesse E, Peplies J, Glöckner FO. SINA: accurate high-throughput multiple sequence alignment of ribosomal RNA genes. Bioinformatics (Oxford, England) 2012;28:1823-1829.

[4] Quast C, Pruesse E, Yilmaz P, Gerken J, Schweer T, Yarza P, et al. The SILVA ribosomal RNA gene database project: improved data processing and web-based tools. Nucleic acids research 2013;41:D590-596.

[5] Davis NM, Proctor DM, Holmes SP, Relman DA, Callahan BJ. Simple statistical identification and removal of contaminant sequences in marker-gene and metagenomics data. Microbiome 2018;6:226.

[6] Thorsen J, Brejnrod A, Mortensen M, Rasmussen MA, Stokholm J, Al-Soud WA, et al. Large-scale benchmarking reveals false discoveries and count transformation sensitivity in 16S rRNA gene amplicon data analysis methods used in microbiome studies. Microbiome 2016;4:62.

[7] Paulson JN, Stine OC, Bravo HC, Pop M. Differential abundance analysis for microbial marker-gene surveys. Nature methods 2013;10:1200-1202.

[8] Moschen AR, Geiger S, Krehan I, Kaser A, Tilg H. Interferon-alpha controls IL-17 expression in vitro and in vivo. Immunobiology 2008;213:779-787.

[9] Schippers IJ, Moshage H, Roelofsen H, Müller M, Heymans HS, Ruiters M, et al. Immortalized human hepatocytes as a tool for the study of hepatocytic (de-)differentiation. Cell Biol Toxicol 1997;13:375-386.

[10] Gervois P, Chopin-Delannoy S, Fadel A, Dubois G, Kosykh V, Fruchart JC, et al. Fibrates increase human REV-ERBalpha expression in liver via a novel peroxisome proliferator-activated receptor response element. Mol Endocrinol 1999;13:400-409.

[11] Ress C, Moschen AR, Sausgruber N, Tschoner A, Graziadei I, Weiss H, et al. The role of apolipoprotein A5 in non-alcoholic fatty liver disease. Gut 2011;60:985-991.
