# Supplementary material for: 24-Norursodeoxycholic acid ameliorates experimental alcohol-related liver disease and activates hepatic PPARγ
Source: JHEP Rep. 2023 Aug 3;5(11):100872. doi: 10.1016/j.jhepr.2023.100872 (PMC10561126; doi:10.1016/j.jhepr.2023.100872)
Supplement: Multimedia component 2 [file mmc2.docx]

**JHEP Reports**

**CTAT methods**

Tables for a “Complete, Transparent, Accurate and Timely account” (CTAT) are now mandatory for all revised submissions. The aim is to enhance the reproducibility of methods.

- Only include the parts relevant to your study
- Refer to the CTAT in the main text as ‘Supplementary CTAT Table’
- Do not add subheadings
- Add as many rows as needed to include all information
- Only include one item per row

**If the CTAT form is not relevant to your study, please outline the reasons why:**

|  |
| --- |

- 1. **Antibodies**

| **Name** | **Citation** | **Supplier** | **Cat no.** | **Clone no.** |
| --- | --- | --- | --- | --- |
| MPO |  | Dako, Santa Clara, CA, USA; | A0398 |  |
| F4/80 |  | Cell Signaling Technology | #70076 |  |
| PPARg |  | Invitrogen | A3409A, |  |
| PPARg |  | Cell Signaling Technology | #2443 |  |
| PPARg |  | Santa-Cruz biotechnology | sc-7273X |  |
| CPT1A |  | Cell Signaling Technology | #97361; |  |
| GAPDH |  | Cell Signaling Technology | #2118 |  |

- 1. **Cell lines**

| **Name** | **Citation** | **Supplier** | **Cat no.** | **Passage no.** | **Authentication test method** |
| --- | --- | --- | --- | --- | --- |
| Human primary immortalized hepatocytes | Schippers IJ et al. Cell Biol Toxicol. 1997; |  |  |  |  |
| HepG2 | Aden D.P et al. Nature. 1979; | ATCC | HB-8065 |  |  |

- 1. **Organisms**

| **Name** | **Citation** | **Supplier** | **Strain** | **Sex** | **Age** | **Overall n number** |
| --- | --- | --- | --- | --- | --- | --- |
| Mus musculus |  | Charles river | C57BL/6 | female | 7-8 week | 100 |

- 1. **Sequence based reagents**

| **Name** | **Sequence** | **Supplier** |
| --- | --- | --- |
|  |  |  |

- 1. **Biological samples**

| **Description** | **Source** | **Identifier** |
| --- | --- | --- |
|  |  |  |

- 1. **Deposited data**

| **Name of repository** | **Identifier** | **Link** |
| --- | --- | --- |
|  |  |  |

- 1. **Software**

| **Software name** | **Manufacturer** | **Version** |
| --- | --- | --- |
| Image Lab software | BioRad |  |
| GraphPad PRISM 5 | La Jolla, California, USA | **5** |

- 1. **Other (*e.g*. drugs, proteins, vectors etc.)**

| norUDCA |  |  |
| --- | --- | --- |
| MCSF | Peprotech |  |
| lipopolysaccharide | Invivogen |  |
| Interferon-*γ* | Peprotech |  |
| Interleukin 4 | Peprotech |  |
| rosiglitazone | Merck |  |

- 1. **Please provide the details of the corresponding methods author for the manuscript:**

| Herbert Tilg, M.D., Department of Internal Medicine I, Medical University Innsbruck, Innsbruck, Austria; Phone: +43 512 504 23539; Fax: +43 512 504 23538; E-mail: herbert.tilg@i-med.ac.at  Michael Trauner, M.D., Department of Medicine III, Medical University of Vienna, Vienna, Austria; Phone: +43 (0)14040047440; Fax: +43 (0)14040047350; E-mail: michael.trauner@meduniwien.ac.at |
| --- |

**2.0 Please confirm for randomised controlled trials all versions of the clinical protocol are included in the submission. These will be published online as supplementary information.**

|  |
| --- |
