# Supplementary material for: 24-Norursodeoxycholic acid ameliorates experimental alcohol-related liver disease and activates hepatic PPARγ
Source: JHEP Rep. 2023 Aug 3;5(11):100872. doi: 10.1016/j.jhepr.2023.100872 (PMC10561126; doi:10.1016/j.jhepr.2023.100872)
Supplement: Multimedia component 4 [file mmc4.pdf]

Research article

JHEP|Reports

24-Norursodeoxycholic acid ameliorates experimental alcohol-related liver disease and activates hepatic PPARγ

Christoph Grander,[1,†] **Moritz Meyer**,[1,†] Daniel Steinacher,[2] Thierry Claudel,[2] Bela Hausmann,[3,4] Petra Pjevac,[3,5] Felix Grabherr,[1] Georg Oberhuber,[6] Manuel Grander,[7] Natascha Brigo,[7] Almina Jukic,[1] Julian Schwärzler,[1] Günter Weiss,[7] Timon E. Adolph,[1] Michael Trauner,[2,*] Herbert Tilg[1,*]

[1]Department of Internal Medicine I, Gastroenterology, Hepatology, Endocrinology and Metabolism, Medical University Innsbruck, Innsbruck, Austria; [2]Hans Popper Laboratory of Molecular Hepatology, Department of Internal Medicine III, Division of Gastroenterology and Hepatology, Medical University of Vienna, Vienna, Austria; [3]Joint Microbiome Facility of the Medical University of Vienna, The University of Vienna, Vienna, Austria; [4]Department of Laboratory Medicine, Medical University of Vienna, Vienna, Austria; [5]Division of Microbial Ecology, Department of Microbiology and Ecosystem Science, Centre for Microbiology and Environmental Systems Science, University of Vienna, Vienna, Austria; [6]INNPATH, Tirol-Kliniken University Hospital Innsbruck, Innsbruck, Austria; [7]Department of Internal Medicine II, Infectious Diseases, Immunology, Rheumatology, Pneumology, Medical University Innsbruck, Innsbruck, Austria

JHEP Reports **2023.** https://doi.org/10.1016/j.jhepr.2023.100872

Background & Aims: Alcohol-related liver disease (ALD) is a global healthcare challenge with limited treatment options. 24-Norursodeoxycholic acid (NorUDCA) is a synthetic bile acid with anti-inflammatory properties in experimental and human cholestatic liver diseases. In the present study, we explored the efficacy of norUDCA in experimental ALD.
Methods: NorUDCA was tested in a preventive and therapeutic setting in an experimental ALD model (Lieber–DeCarli diet enriched with ethanol). Liver disease was phenotypically evaluated using histology and biochemical methods, and anti-inflammatory properties and peroxisome proliferator-activated receptor gamma activation by norUDCA were evaluated in cellular model systems.
Results: NorUDCA administration ameliorated ethanol-induced liver injury, reduced hepatocyte death, and reduced the expression of hepatic pro-inflammatory cytokines including *tumour necrosis factor* (*Tnf*), *Il-1β*, *Il-6*, and *Il-10*. NorUDCA shifted hepatic macrophages towards an anti-inflammatory M2 phenotype. Further, norUDCA administration altered the composition of the intestinal microbiota, specifically increasing the abundance of *Roseburia*, *Enterobacteriaceae*, and *Clostridum* spp. In a therapeutic model, norUDCA also ameliorated ethanol-induced liver injury. Moreover, norUDCA suppressed lipopolysaccharide-induced IL-6 expression in human peripheral blood mononuclear cells and evoked peroxisome proliferator-activated receptor gamma activation.
Conclusions: NorUDCA ameliorated experimental ALD, protected against hepatic inflammation, and affected gut microbial commensalism. NorUDCA could serve as a novel therapeutic agent in the future management of patients with ALD.
Impact and implications: Alcohol-related liver disease is a global healthcare concern with limited treatment options. 24-Norursodeoxycholic acid (NorUDCA) is a modified bile acid, which was proven to be effective in human cholestatic liver diseases. In the present study, we found a protective effect of norUDCA in experimental alcoholic liver disease. For patients with ALD, norUDCA could be a potential new treatment option.
© 2023 The Authors. Published by Elsevier B.V. on behalf of European Association for the Study of the Liver (EASL). This is an open access article under the CC BY license (http://creativecommons.org/licenses/by/4.0/).

Introduction

Alcohol overconsumption is estimated to cause 5.3% of all deaths worldwide[1,2] and is still the most common indication for liver transplantation in Europe.[3] The hepatic manifestation of alcohol overconsumption is alcohol-related liver disease (ALD), comprising a spectrum from hepatic steatosis, hepatitis (alcoholic hepatitis) to fibrosis, cirrhosis, and hepatocellular carcinoma. The pathogenesis of ALD is multilayered and involves hepatic ethanol toxicity resulting in the generation of reactive oxygen species,[4] as well as the increased translocation of pathogen-associated molecular patterns (such as lipopolysaccharide [LPS]) from the leaky gut into the systemic circulation. Ethanol consumption has been shown to alter the intestinal microbiota, leading to a decrease in gut barrier function.[5–8] Furthermore, alcohol consumption perturbates bile acid metabolism.[9,10] For example, ethanol downregulates farnesoid X receptor (FXR/NR1H4),[11,12] alters bile acid conjugation,[13] and affects enterohepatic bile acid circulation.[13,14] Chronic alcohol consumption results in an increased bile acid pool and

Keywords: Alcoholic liver disease; norUDCA; Microbiota; M2 macrophages; Ppar-gamma.
Received 13 December 2022; received in revised form 23 May 2023; accepted 12 July 2023; available online 3 August 2023
† These authors contributed equally.
* Corresponding authors. Addresses: Department of Internal Medicine I, Medical University Innsbruck, Innsbruck, Austria. Tel.: +43-512-504-23539; Fax: +43-512-504-23538 (H. Tilg); Department of Medicine III, Medical University of Vienna, Vienna, Austria. Tel.: +43-(0)14040047440; Fax: +43-(0)14040047350 (M. Trauner).
E-mail addresses: herbert.tilg@i-med.ac.at (H. Tilg), michael.trauner@meduniwien.ac.at (M. Trauner).

ELSEVIER

EASL The Home of Hepatology

decreased excretion of bile acids. Increased exposure to toxic bile acids might thereby aggravate hepatic injury.[14] All these factors facilitate the production of pro-inflammatory cytokines such as IL-6, tumour necrosis factor (TNF), and IL-1β along with the infiltration of pro-inflammatory cells,[15] which culminates in cell necrosis, liver injury, and fibrogenesis.[16,17]

In addition to inflammatory mediators, numerous immune cell populations are involved in the pathogenesis of ALD. Studies have shown that infiltrating macrophages polarise in response to microenvironmental signals and play a crucial role in pathophysiological processes such as inflammation, tumour development, tissue repair, and metabolism.[15,18–20] Ethanol is known to promote the polarisation of M1 macrophages through NF-κB signalling.[21] Peroxisome proliferator-activated receptor gamma (PPARg/NR1C3) is a nuclear receptor inhibiting the expression of inflammatory cytokines and inducing the differentiation of immune cells towards an anti-inflammatory (M2) phenotype.[22–24]

24-Norursodeoxycholic acid (NorUDCA) is a side chain-shortened ursodeoxycholic acid (UDCA) with potent choleretic properties.[25,26] Owing to the relative resistance of norUDCA to conjugation, it is reabsorbed by cholangiocytes and shunted between bile ducts and hepatocytes, resulting in increased hepatic exposure and secretion of biliary bicarbonate shielding the bile epithelia from bile acid toxicity.[25–27] NorUDCA ameliorated the pathology in *Mdr2*-deficient mice, a model of primary sclerosing cholangitis (PSC),[28,29] and showed potent anti-inflammatory effects in mouse models of non-cholestatic liver injury such as hepatic schistosomiasis,[28] experimental non-alcoholic steatohepatitis,[27] and acute non-cytolytic lymphocytic choriomeningitis virus infection.[30] Importantly, when compared with placebo, norUDCA was effective in reducing serum levels of alkaline phosphatase and transaminases in patients with PSC.[31] Furthermore, in a recent placebo-controlled clinical trial,[32] treatment of patients with non-alcoholic fatty liver disease (NAFLD) with norUDCA resulted in a dose-dependent reduction of serum alanine aminotransferase (ALT) within 12 weeks, suggesting efficacy in human NAFLD.

Here, we explored the therapeutic efficacy of norUDCA in experimental ALD. NorUDCA treatment ameliorated experimental liver disease, as demonstrated by reduced liver injury, reduced hepatocyte death, and reduced expression of pro-inflammatory cytokines. NorUDCA enhanced PPARg activity independent from ethanol exposure and increased abundance of potentially beneficial bacteria.

Materials and methods

Mouse experiments

Two different models of experimental ALD were used to study the role of norUDCA in ALD. All experiments were aligned to ethical principles according to Austrian laws (2020-0.152.544) and were carried out in the animal facility of the Medical University of Innsbruck. (1) To study the effect of norUDCA in a preventive setting, 7- to 8-week-old female wild-type (wt/wt; C57BL/6) mice were fed a Lieber–DeCarli diet containing 1–5 vol% (ethanol-fed) *ad libitum* for 15 days (Safe, Rosenberg, Germany). Control groups were fed a pair diet containing isocaloric maltose. Half of the ethanol-fed and pair-fed groups were treated with norUDCA (1 mg/ml diet, 5% wt/wt[30]) by supplementing norUDCA into the diet. (2) To study possible therapeutic effects of norUDCA in experimental ALD, 7- to 8-week-old female wild-type mice were fed a Lieber–DeCarli diet containing 1–5 vol% (ethanol-fed) *ad libitum* for 15 days. NorUDCA treatment was conducted from Day 10 to Day 15.

Histology

Liver tissue samples were fixed in formaldehyde (Sigma, St. Louis, MO, USA) immediately after sacrifice. Samples were embedded in paraffin and further prepared at the Department of Pathology at the Medical University of Innsbruck. Liver sections were deparaffinised in xylene and dehydrated in an ethanol gradient. Dehydration, embedding, and H&E staining[33] were carried out at the Department of Pathology at the Medical University of Innsbruck, before stained slides were evaluated by an experienced pathologist (GO, INNPATH). Up to 20 high-power fields (1 mm^2) per slide were analysed.

Microbiome studies

The microbial community composition in collected caecal stool samples was analysed by ribosomal small subunit (SSU rRNA/16S rRNA) gene amplicon sequencing[34] at the Joint Microbiome Facility (Vienna) under the Project ID at the Joint Microbiome Facility (Vienna). DNA extraction from stool samples using the QIAamp DNA Fast stool Kit was automated on the QiaCube Connect. For microbial community profiling, the 16S rRNA genes were amplified by PCR applying primers that cover most bacterial and archaeal clades (515F, 806R).[35] After PCR amplification of the marker gene region, the amplicons were barcoded, multiplexed, and sequenced on the Illumina MiSeq platform at the Joint Microbiome Facility.[34] Negative controls were performed during sampling, DNA extraction, and barcoding. Further details on amplicon sequence data processing are provided in the Supplementary methods.

Data availability

SSU rRNA gene amplicon datasets are deposited in Sequence Read Archive (SRA) under the BioProject accession number PRJNA907184.

Statistical analysis

For analysing the present data, we used GraphPad Prism 5 (La Jolla, CA, USA). Unpaired two-tailed Student's *t* test, the Kruskal–Wallis test followed by Dunn's multiple comparison test, and one-way ANOVA followed by the *post hoc* Newman–Keuls test were used where appropriate. Two or more independent experiments were performed for each modality. Results are shown as mean ± SEM. Statistical significance was considered at $p < 0.05$.

Further information on materials and methods are provided in the Supplementary materials.

Results

NorUDCA treatment protects from experimental ALD

Mice were fed an ethanol-enriched diet with/without norUDCA (1 mg/ml, 5% wt/wt) supplementation for 15 days (Fig. 1A). Ethanol feeding resulted in liver injury, as indicated by significantly increased ALT levels ($p < 0.001$; Fig. 1B) and an increased number of intrahepatic terminal deoxynucleotidyl transferase dUTP nick end labelling-positive ($TUNEL^+$) apoptotic hepatocytes, when compared with that in pair-fed control mice (Fig. 1C and D). NorUDCA supplementation reduced ALT levels ($p < 0.001$; Fig. 1B) and the number of $TUNEL^+$ cells ($p < 0.05$; Fig. 1C and D) in ethanol-fed mice. Serum ethanol concentrations were not significantly different between ethanol-fed groups (Fig. S1A), but mRNA expression of ethanol-metabolising enzymes such as

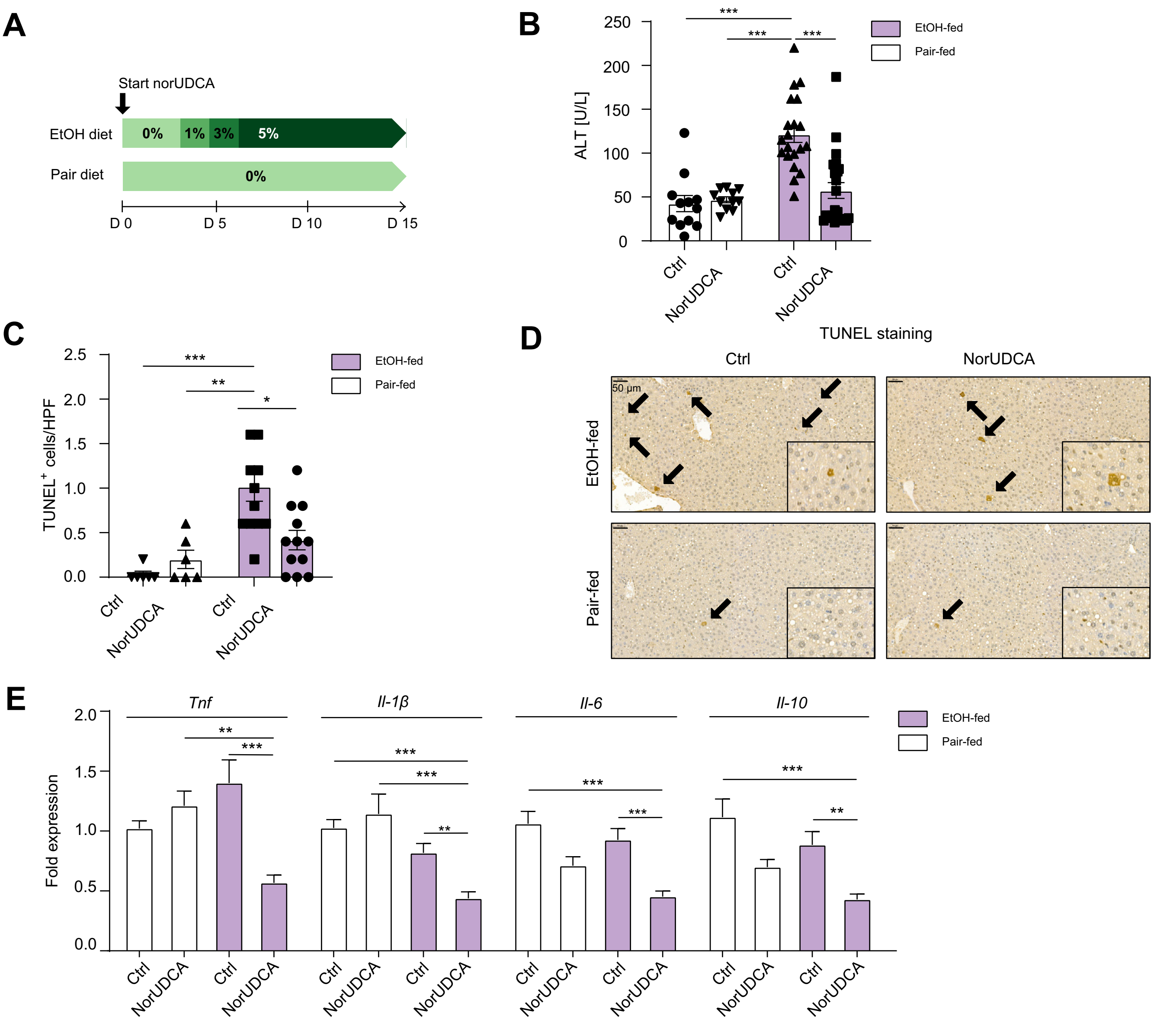

Fig. 1. NorUDCA protects from experimental alcoholic liver disease. (A) Graphical illustration of experimental design. (B) Serum concentrations of ALT and (C) number of TUNEL$^+$ cells were significantly decreased in norUDCA-treated, EtOH-fed mice compared with Ctrl. (D) Representative images and quantification of TUNEL$^+$ liver cells per HPF based on immunoreactivity (brown indicates TUNEL$^+$ cells, black arrows indicate positivity of TUNEL+ cells). (E) NorUDCA treatment significantly decreased the mRNA expression of *Tnf*, *Il-1β*, *Il-6*, and *Il-10* in EtOH-fed mice compared with house-keeping gene β-actin. Data are shown as mean ± SEM. *p <0.05, **p <0.01, and ***p <0.001 according to one-way ANOVA with Bonferroni *post hoc* analysis or the Kruskal–Wallis test with Dunn's *post hoc* analysis. β-actin was used as a housekeeping gene (E). ALT, alanine aminotransferase; Ctrl, control; EtOH, ethanol; HPF, high-power field; norUDCA, 24-norursodeoxycholic acid; *Tnf*, tumour necrosis factor; TUNEL$^+$, terminal deoxynucleotidyl transferase dUTP nick end labelling-positive.

alcohol dehydrogenase-1 (*Adh-1*) (p <0.01; Fig. S1B), *Adh-5* (p <0.001; Fig. 1C), aldehyde dehydrogenase-1a1 (*Aldh-1a1*) (p <0.001; Fig. S1D), and *Aldh-4a1* (p <0.001; Fig. S1E) was significantly increased in norUDCA-treated mice compared with ethanol-fed controls. To further investigate the influence of norUDCA on ethanol-induced hepatic injury, we analysed the expression of pro-inflammatory cytokines. NorUDCA reduced the hepatic mRNA expression of *Tnf* (p <0.001), *Il-1β* (p <0.01), *Il-6* (p <0.001), and *Il-10* (p <0.01) compared with that in ethanol-fed controls (Fig. 1E).

M2 macrophage polarisation is induced by norUDCA

In a next step, the number of hepatic macrophages was quantified by F4/80 staining. Ethanol feeding tended to increase the numbers of hepatic F4/80$^+$ cells (p = 0.104; Fig. 2A and B), whereas norUDCA treatment significantly increased the number of hepatic F4/80$^+$ cells in both ethanol-fed (p <0.001; Fig. 2A and B) and pair-fed mice (p <0.05; Fig. 2A and B). In norUDCA-treated, ethanol-fed mice, hepatic macrophage composition predominantly consisted of anti-inflammatory M2 polarised macrophages, as indicated by a decreased hepatic expression of

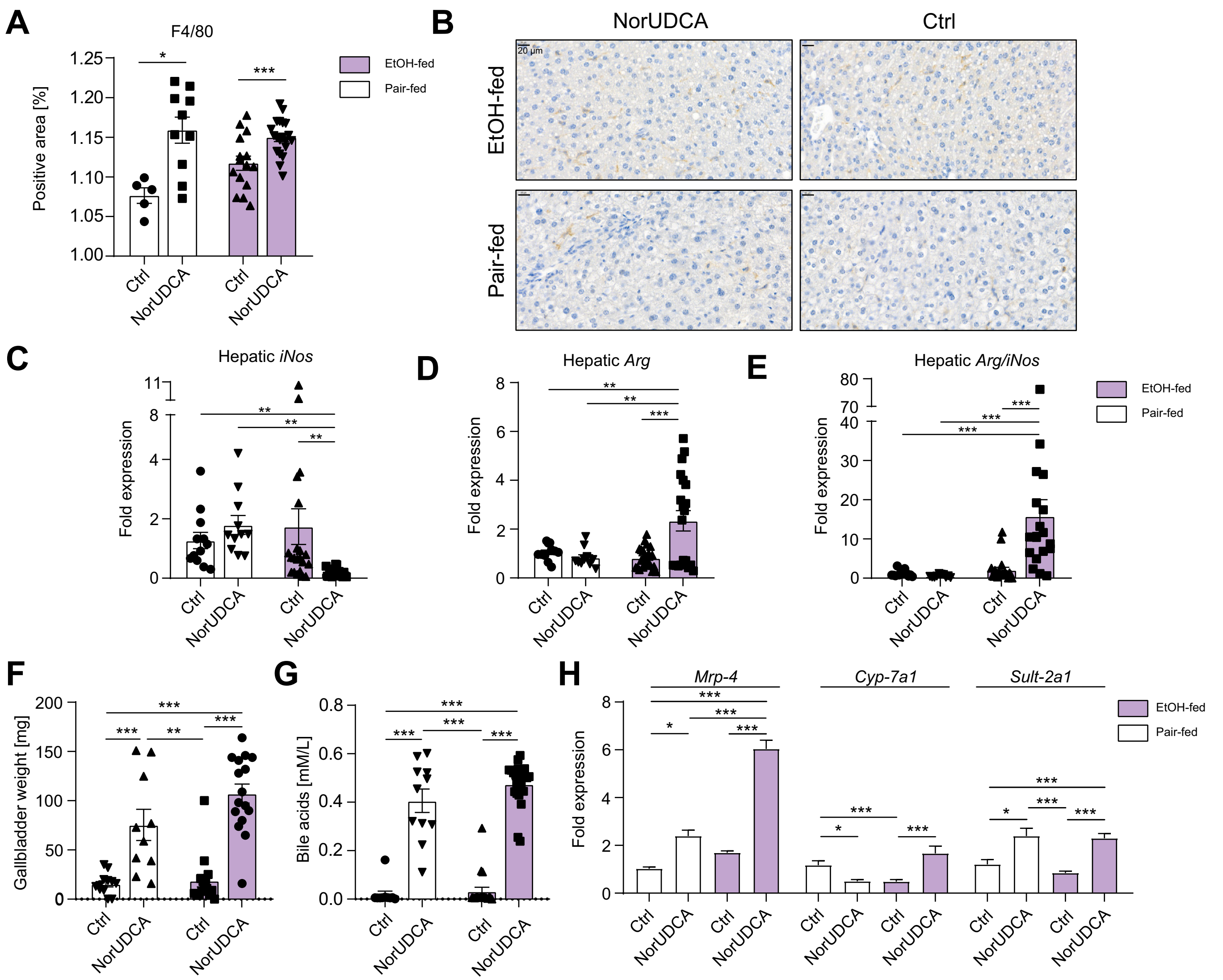

Fig. 2. NorUDCA induces a M2 hepatic macrophage phenotype. (A) Quantification of F4/80⁺-positive area per high-power field based on F4/80 immunoreactivity. (B) Representative images of F4/80⁺-stained liver sections (brown indicates F4/80⁺ cells). Pronounced M2 macrophage phenotype could be observed in norUDCA-treated, EtOH-fed mice, indicated by (C) significantly decreased hepatic *iNos* and (D) significantly increased Arg expression as well as (E) *Arg*/*iNos* ratio. (F) Gallbladder weight and (G) serum bile acid concentrations were increased in norUDCA-treated mice in pair-fed and EtOH-fed mice. (H) NorUDCA treatment significantly altered the hepatic expression of proteins involved in bile acid metabolism such as *Mrp4* (left), *Cyp7a1* (middle), and *Sult2a1* (right). Data are shown as mean ± SEM. *p <0.05, **p <0.01, and ***p <0.001 according to one-way ANOVA with Bonferroni *post hoc* analysis or the Kruskal–Wallis test with Dunn's *post hoc* analysis. β-actin was used as a housekeeping gene (C–E, H). *Arg*, arginase; Ctrl, control; *Cyp7a1*, cytochrome P450 family 7 subfamily A member 1; EtOH, ethanol; *iNos*, inducible nitric oxide synthase; *Mrp4*, multidrug resistance-associated protein 4; norUDCA, 24-norursodeoxycholic acid; *Sult2a1*, sulfotransferase family 2A member 1.

inducible nitric oxide synthase (*iNos*) (p <0.01; Fig. 2C) and an increased expression of arginase (*Arg*) (p <0.001; Fig. 2D), *Arg*/*iNos* ratio (p <0.001; Fig. 2E), and *Cd206*/*iNos* ratio (Fig. S2A and B). To further evaluate this finding *in vitro*, we performed FACS analysis of murine bone marrow derived macrophages after stimulation with norUDCA, LPS, IL-4, and interferon gamma.[36] NorUDCA treatment significantly shifted the relative number of M1/M2 in favour of M2 macrophages compared with that in LPS-stimulated controls (p <0.001; Fig. S2C–G). Ethanol feeding resulted in increased infiltration of myeloperoxidase-positive neutrophils (Fig. S2H and I).

NorUDCA affects bile acid metabolism

As expected, norUDCA treatment altered bile acid metabolism. An increased gallbladder weight could be observed in both pair-fed (p <0.001; Fig. 2F) and ethanol-fed mice (p <0.001; Fig. 2F) treated with norUDCA, in line with its potent choleretic effects. Similarly, plasma bile acid concentration was significantly higher in norUDCA-treated mice in both the pair-fed (p <0.001; Fig. 2G) and ethanol-fed (p <0.001; Fig. 2G) groups, whereas no difference in gallbladder weight or bile acid concentration could be observed between control groups (Fig. 2F and G). Furthermore, norUDCA significantly increased the hepatic mRNA expression of

enzymes involved in the regulation of bile acid synthesis, metabolism, and export, including multidrug resistance-associated protein 4 *(Mrp4)* (*p* <0.001; Fig. 2H, left panel), cytochrome P450 family 7 subfamily A member 1 (*Cyp7a1*) (*p* <0.001; Fig. 2H, middle panel), sulfotransferase family 2A member 1 *(Sult2a1)* (*p* <0.001; Fig. 2H, right panel), and acyl-CoA oxidase 1 *(Acox1)* (*p* <0.001; Fig. S1F).

NorUDCA impacts on hepatic lipid metabolism

Hepatic steatosis was quantified on H&E-stained liver slides by an experienced board-certified liver pathologist based on a previously established scoring system.[37] Ethanol feeding led to enhanced hepatic lipid accumulation (*p* <0.001; Fig. 3A and B), whereas norUDCA treatment significantly decreased the hepatic steatosis score in ethanol-fed mice (*p* <0.001; Fig. 3A and B). NorUDCA mainly tended to reduce microvesicular and mediovesicular hepatic steatosis in ethanol-fed mice (Fig. S3A and B), whereas macrovesicular steatosis was unchanged by norUDCA treatment compared with that in ethanol-fed controls (Fig. S3C). This could explain the tendency towards increased accumulation of triglycerides in the liver of ethanol-fed, norUDCA-treated mice compared with controls (Fig. 3C). In a next step, we performed quantitative PCR analysis of several genes involved in hepatic fatty acid metabolism. Interestingly, in ethanol-fed mice, norUDCA significantly increased the hepatic expression of *Pparg* (*p* <0.001; Fig. 3D), carnitine palmitoyltransferase 1 (*Cpt-1*) (*p* <0.01; Fig. 3D), and sterol regulatory element-binding protein 1 *(Srebp1c)* (*p* <0.001; Fig. 3D).

NorUDCA shows potent anti-inflammatory properties via PPARg activation

PPARg (mainly isoform PPARg1) levels were increased upon norUDCA administration in ethanol-fed mice (Fig. 3E and F). As PPARg was shown to exhibit anti-inflammatory properties and to induce an anti-inflammatory (M2) macrophage phenotype, we assessed the activation of PPARg upon norUDCA stimulation. Using human immortalised hepatocytes and a luciferase assay, we could demonstrate an activation of PPARg by norUDCA (Fig. S3D). In a second step, immortalised human hepatocytes were stimulated with norUDCA (500 μM), rosiglitazone (PPARg agonist, 10 μM), and control for 48 h. Notably, PPARg was enhanced after norUDCA stimulation comparable with rosiglitazone in nuclear extracts (Fig. 3G). To further study the anti-inflammatory potential of norUDCA as indicated by a decreased expression of pro-inflammatory cytokines and an increased *Pparg* expression, we performed an *in vitro* experiment, stimulating peripheral blood mononuclear cells (PBMCs) of healthy donors with LPS and norUDCA (50 and 500 μM) for 24 h. IL-6 levels were significantly decreased in PBMCs stimulated with 500 μM norUDCA (*p* <0.05; Fig. 3H). Furthermore, we observed a trend towards increased *Pparg* expression in HepG2 cells upon norUDCA and LPS treatment (Fig. S3E).

NorUDCA treatment alters intestinal microbiota composition

In a next step, we evaluated the influence of norUDCA on the intestinal microbiota composition. In weighted UniFrac principal coordinates analysis plots, a significant dissimilarity between all four investigated groups was observed (permutational multivariate ANOVA, *p* <0.001; Fig. 4A). At the genus level, in both norUDCA-treated mice groups, we observed a decrease of *Muribaculaceae*- and *Fecalibactulum*-related Amplicon Sequence Variants (ASVs), compared with that in controls. *Roseburia*-, *Enterobacteriaceae*-, and *Clostridium*-related ASVs, by contrast, were relatively more abundant in norUDCA-treated mice (Fig. 4B). Moreover, norUDCA supplementation upregulated the expression of ileal claudin 4 (*Cldn-4*) (*p* <0.05; Fig. 4C) and tight junction protein 1 (*Tjp-1*) (*p* <0.01; Fig. 4C) in ethanol-fed mice. In the colon, norUDCA treatment significantly increased the expression of *Cldn-4* in pair-fed mice (*p* <0.01; Fig. 4D) as well as in ethanol-fed mice (*p* <0.001; Fig. 4D), which might reflect improved gut integrity. Ethanol-induced increase of circulating LPS tended to be lower in ethanol-fed, norUDCA-treated mice than in controls (Fig. S3F). LPS-binding protein (*LBP*) expression was also not different within the groups (Fig. S3G).

Therapeutic norUDCA treatment ameliorates experimental ALD

To further assess the potential therapeutic effect of norUDCA in already established experimental ALD, mice were fed a Lieber–DeCarli diet for 15 days, and norUDCA supplementation was started at Day 10 (Fig. 5A). Ethanol feeding resulted in significantly increased ALT levels compared with that in pair-fed control mice, whereas norUDCA treatment decreased the ALT by 45% in ethanol-fed mice (*p* = 0.06; Fig. 5B). Serum ethanol concentration was not significantly different between the groups (Fig. S4A). Gallbladder weight, as a surrogate marker for increased bile flow, was significantly higher in norUDCA-treated mice in both the ethanol- and pair-fed groups (Fig. S4B). Eventually, the hepatic expression of *Mrp4* was increased in ethanol-fed mice, but not pair-fed mice, upon norUDCA administration (Fig. S4C). NorUDCA increased the hepatic expression of *Pparg* (*p* <0.001; Fig. 5C) and *Cpt-1* (*p* <0.001; Fig. 5C) in both pair-fed and ethanol-fed mice, whereas norUDCA decreased fatty acid synthase (*Fasn*) expression only in ethanol-fed mice (*p* <0.01; Fig. 5C). On the protein level, CPT-1 was augmented in ethanol-fed, norUDCA-treated mice (Fig. S4D and E).

NorUDCA induced a reduction of hepatic *Tnf* (*p* <0.05) and *Il-6* (*p* <0.05) expression in ethanol-fed mice (Fig. 5D). Similar to that in the preventive setting, norUDCA treatment increased the number of F4/80$^+$ cells in ethanol- and pair-fed mice (Fig. 5E and F). Myeloperoxidase-positive neutrophils were increased by ethanol feeding compared with those in controls but were lowered upon norUDCA treatment in ethanol-fed mice (*p* <0.001; Fig. 5G and H).

Discussion

We investigated the efficacy of norUDCA in experimental ALD. Although ALD is one of the most frequent liver diseases, with alcohol-related liver cirrhosis being associated with 22.2 million disability adjusted life years in 2016, therapeutic options are still limited.[38] Different mechanisms contribute to the development of ALD, including ethanol toxicity, gut barrier dysfunction, and endotoxaemia.[5,6,8,39] Notably, bile acids may also play an important role in the pathogenesis of ALD.[9]

In this study, we demonstrated that the administration of norUDCA protected wild-type mice from ethanol-induced liver injury in a preventive and therapeutic setting. Furthermore, we unravelled several potential mechanisms how norUDCA, a side chain shortened homologue of UDCA, ameliorates experimental ALD. Specifically, we demonstrated that norUDCA treatment ameliorated serum concentrations of ALT, reduced hepatic cell death, and decreased hepatic expression of pro-inflammatory cytokines, such as *Tnf*, *Il-6*, and *Il-1β*. The amelioration of pro-

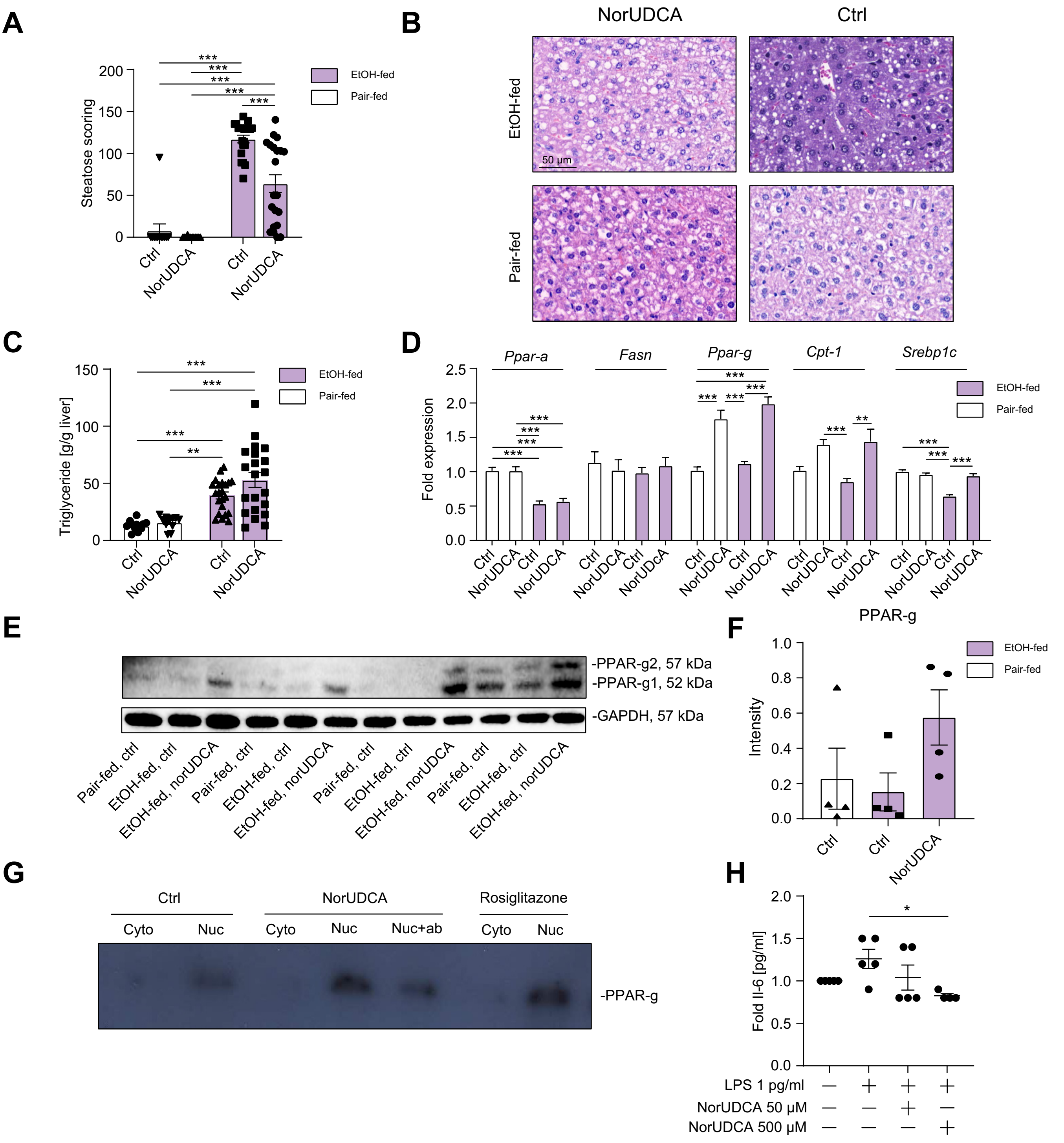

Fig. 3. NorUDCA alters hepatic lipid metabolism by induction of PPARg. (A) NorUDCA treatment was associated with decreased hepatic steatosis scoring in EtOH-fed mice. (B) Representative H&E-stained liver sections. (C) EtOH feeding resulted in increased triglyceride accumulation, although no significant difference between EtOH-fed, norUDCA-treated mice and Ctrl could be observed. (D) NorUDCA treatment significantly increased the hepatic expression of *Pparg* in both EtOH-fed and pair-fed mice, and *Cpt-1* and *Srebp1c* in EtOH-fed mice. (E) Western blot analysis of PPARg1/2 and GAPDH and (F) quantification of PPARg1 and PPARg2. (G) Human primary immortalised hepatocytes were stimulated with norUDCA (500 μM), rosiglitazone (PPARg agonist, 10 μM), and Ctrl. PPARg was enhanced after norUDCA stimulation comparable with rosiglitazone in nuc. To confirm correct detection, PPARg antibody (nuc + ab) was used to decrease binding capability of the assay. (H) Human PBMCs were treated with LPS (1 pg/ml) and norUDCA (50 and 500 μM). IL-6 was measured after 24 h in the supernatant. NorUDCA decreased LPS-induced IL-6 response. Data are shown as mean ± SEM. *p <0.05, **p <0.01, and ***p <0.001 according to one-way ANOVA with Bonferroni *post hoc* analysis or the Kruskal–Wallis test with Dunn's *post hoc* analysis. β-actin was used as a housekeeping gene (D). Ctrl, control; *Cpt-1*, carnitine palmitoyltransferase 1; cyto, cytosol; EtOH, ethanol; *Fasn*, fatty acid synthetase; GAPDH, glyceraldehyde-3-phosphate dehydrogenase; LPS, lipopolysaccharide; norUDCA, 24-norursodeoxycholic acid; nuc, nuclear extracts; PBMC, peripheral blood mononuclear cell; *Pparg*, peroxisome proliferator-activated receptor gamma; *Srebp1c*, sterol regulatory element-binding protein 1c.

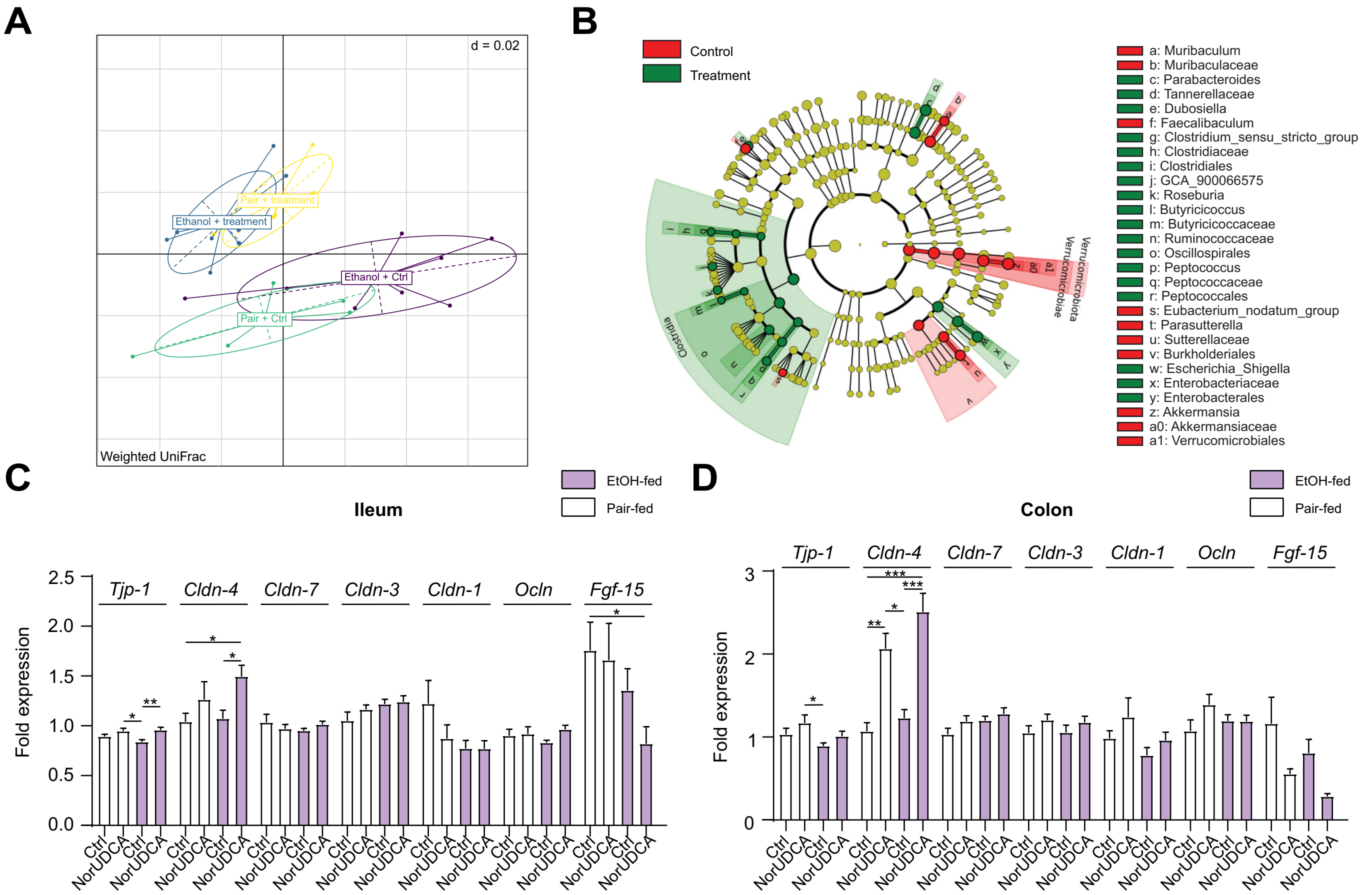

Fig. 4. NorUDCA alters intestinal microbiota composition. (A) NorUDCA altered intestinal microbiota composition displayed by significant differences between groups in the principal coordinates analysis. (B) Analysis of relative abundances using LEfSe, indicating that multiple taxa are differentially relatively abundant in the caecal content of norUDCA-treated mice compared with Ctrl. (C) Ileal and (D) colonic expression of *Cldn-4* was increased by norUDCA in EtOH- and pair-fed mice. Data are shown as mean ± SEM. **p* <0.05, ***p* <0.01, and ****p* <0.001 according to one-way ANOVA with Bonferroni *post hoc* analysis or the Kruskal–Wallis test with Dunn's *post hoc* analysis. β-actin was used as a housekeeping gene (C and D). Ctrl, control; *Cldn*, claudin; EtOH, ethanol; *Fgf-15*, fibroblast growth factor-15; *Tjp-1*, tight junction protein 1; norUDCA, 24-norursodeoxycholic acid; Ocln, ocludin.

inflammatory pathways could potentially be explained by an increased expression of anti-inflammatory *Pparg* in norUDCA-treated mice. In addition, norUDCA could alter the intestinal microbiota in both ethanol- and pair-fed mice.

NorUDCA, was primarily tested in the treatment of cholestatic liver diseases such as PSC. In an experimental PSC model, *Mdr2*$^{-/-}$ mice treated with norUDCA showed reduced hepatic inflammation.[25] Furthermore, norUDCA was proven to affect cells of the innate and adaptive immune system such as macrophages and CD8^{+} T cells in models of experimental cholestatic liver diseases.[30] In hepatocyte-specific NF-κB essential modulator (NEMO)-deficient mice, a genetic model for NAFLD, norUDCA attenuated liver damage, depicted by decreased transaminases, histological improvement, and reduced hepatic fibrosis.[27] The anti-inflammatory properties of norUDCA were further observed in a model of *Schistosoma mansoni*-induced liver injury, whereas norUDCA treatment attenuated liver inflammation by reducing the expression of MHC class II molecules on antigen-presenting cells including macrophages.[28]

Likewise, in ALD, macrophages represent a cornerstone in the pathogenesis of the disease.[40] Under numerous stimulating factors in their microenvironment, macrophages may polarise into either pro-inflammatory M1 or anti-inflammatory M2 phenotype. Whereas the macrophage phenotype M1 is induced by LPS and interferon gamma, IL-13 and IL-4 activate M2 macrophages.[41] Ethanol administration likewise induces the polarisation of M1 macrophages through NF-κB signalling.[20,21,42]

In our study, we found increased numbers of F4/80^{+} macrophages after norUDCA administration in ethanol-fed mice as well as in pair-fed mice. Interestingly, macrophages showed a pronounced M2 phenotype depicted by an increased *Arg/iNos* expression only in norUDCA-treated, ethanol-fed mice. We were further able to demonstrate the anti-inflammatory properties of norUDCA on macrophages *in vitro*. NorUDCA treatment attenuated pro-inflammatory cytokine production upon LPS stimulation of PBMCs. In conclusion, we could show an amelioration of the ethanol-induced liver injury by norUDCA, possibly mediated by enhanced macrophage M2 polarisation.

PPARg is a nuclear receptor with potent effects in metabolic and inflammatory pathways that may be present in different isoforms,[43] namely, PPARg1 and PPARg2. Whereas PPARg1 shows anti-inflammatory properties and is expressed in macrophages,[44,45] PPARg2 is mainly involved in lipid storage and is found in adipocytes as well as in hepatocytes.[46] In the course of

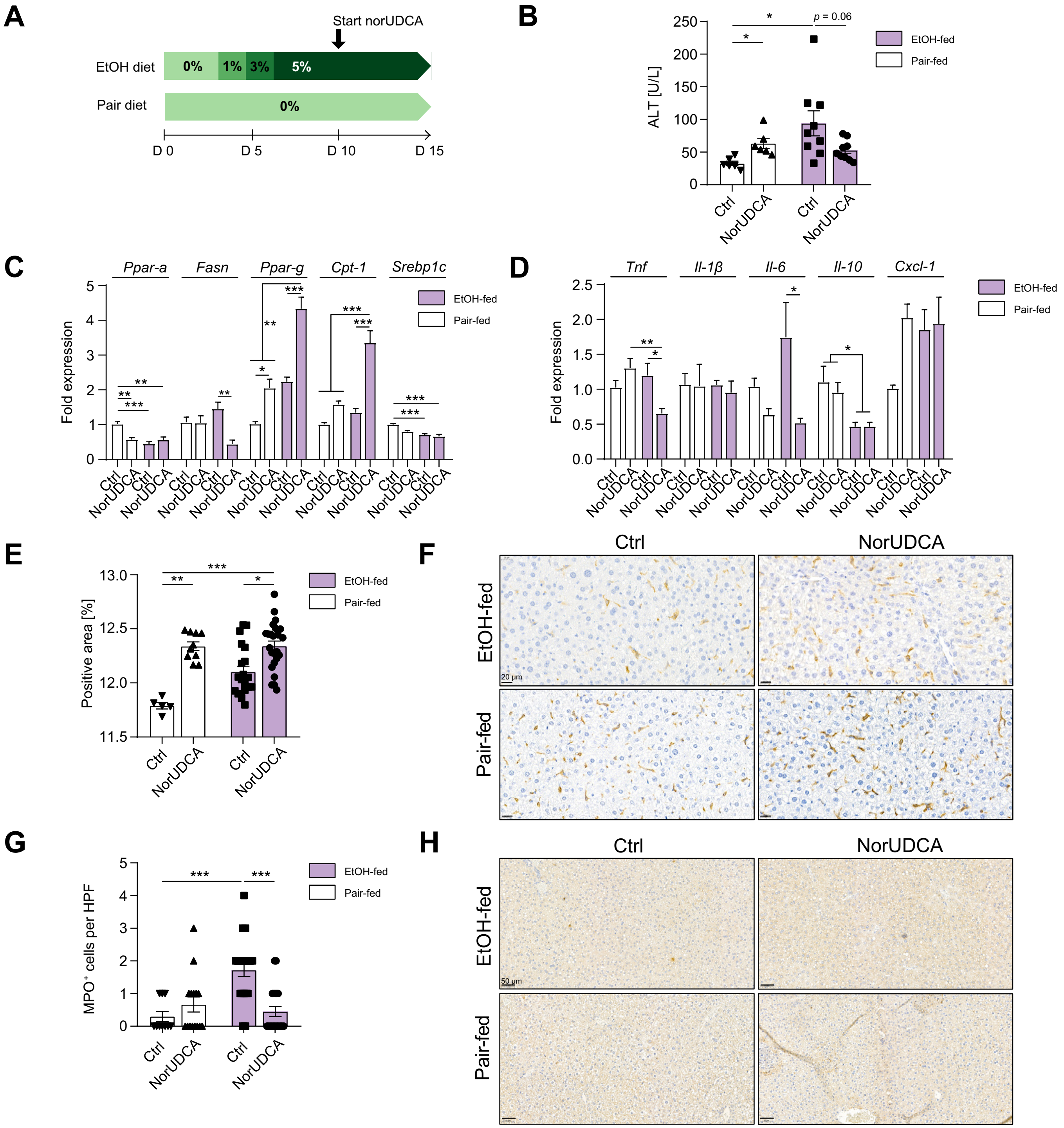

Fig. 5. Therapeutic norUDCA supplementation ameliorates experimental ALD. (A) Graphical illustration of experimental design. (B) ALT concentration tended to be decreased upon norUDCA in EtOH-fed mice. (C) Hepatic expression of *Fasn*, *Pparg*, and *Cpt-1* was altered by norUDCA. (D) Hepatic expression of pro-inflammatory cytokines including *Tnf*, *Il-6*, and *Il-10* was decreased by norUDCA in EtOH-fed mice. (E) Quantification of F4/80$^+$-positive area per HPF based on F4/80 immunoreactivity. (F) Representative images of F4/80$^+$-stained liver sections (brown indicates F4/80$^+$ cells). (G) Number of MPO$^+$ cells was decreased in norUDCA-treated, EtOH-fed mice compared with Ctrl. (H) Representative images of MPO-stained liver sections (brown indicates MPO$^+$ cells). Data are shown as mean ± SEM. $^*p$ <0.05, $^{**}p$ <0.01, and $^{***}p$ <0.001 according to one-way ANOVA with Bonferroni *post hoc* analysis or the Kruskal–Wallis test with Dunn's *post hoc* analysis. β-actin was used as a housekeeping gene (C and D). ALT, alanine aminotransferase; *Cpt-1*, carnitine palmitoyltransferase 1; ALD, alcohol-related liver disease; Ctrl, control; *Cxcl-1*, C-X-C motif chemokine ligand 1; EtOH, ethanol; *Fasn*, fatty acid synthetase; HPF, high-power field; MPO, myeloperoxidase; norUDCA, 24-norursodeoxycholic acid; *Ppar*, peroxisome proliferator-activated receptor; *Srebp1c*, sterol regulatory element-binding protein 1c; *Tnf*, tumour necrosis factor.

exploring new therapeutic agents for fatty liver disease, PPARg agonists (such as rosiglitazone) were tested. Different studies found improved metabolic parameters and antisteatogenic effects in NAFLD upon rosiglitazone treatment, serving as a potent inducer of PPARg.[47–50] Although chronic ethanol consumption was associated with an activation of PPARg2,[51] PPARg2$^{-/-}$ mice had decreased ethanol-induced liver injury.[52] Nevertheless, the role of PPARg1 in ALD is currently unclear.

Notably, in our study, we found a significantly enhanced expression of *Pparg* upon norUDCA administration, both in the preventive as well as therapeutic setting and in pair-fed as well as ethanol-fed mice. Moreover, we could demonstrate a strong induction of anti-inflammatory PPARg1 in livers of norUDCA-treated, ethanol-fed mice. The induction of PPARg by norUDCA was further confirmed by two different *in vitro* assays. Interestingly, glitazones were tested in a clinical trial to treat alcohol addiction,[53] but the trial was stopped early because of increased craving in pioglitazone-treated patients as a result of augmented neuroendocrine stress response to LPS. In line with these data, PPARg agonists were not influencing IL-6 and TNF levels in macrophages *in vitro* or *in vivo*.[54] Therefore, an alternative strategy to target PPARg without facing the adverse effects of its synthetic ligands could be norUDCA.

Similarly, Beraza *et al.*[27] observed tendency towards increased *Pparg* expression after UDCA administration compared with that in controls, whereas norUDCA decreased the expression in *NEMO*$^{-/-}$ mice. Interestingly, PPARg is an important regulator of both cell differentiation and polarisation of macrophages induced by NF-κB and IL-4/IL-13,[20,22,55] which also might explain the increase of anti-inflammatory M2 macrophage polarisation in our study. Wagner *et al.*[56] found improved endothelial barrier and reduced inflammatory parameters in mice treated with ethanol, LPS and rosiglitazone compared to ethanol- and LPS-treated controls. In conclusion, norUDCA induces PPARg activation, and this effect might contribute to its anti-inflammatory mode of action in the liver, mainly by ameliorating the hepatic cytokine response and induction of an M2 macrophage phenotype.

Changes within the intestinal microbiota composition and alcohol-induced liver disease are closely associated. Alcohol overconsumption results in microbial changes, but different bacterial strains may influence disease progression. In weighted UniFrac principal coordinates analysis plots, all four observed groups were significantly different, suggesting that not only ethanol feeding but also norUDCA treatment changed the intestinal microbiota composition in our experiments. Comparing norUDCA treatment groups with controls, we could observe a decreased abundance of *Muribaculaceae*- and *Fecalibactulum*-related ASVs. *Muribaculaceae,* a not well-described strain, was increased by UDCA treatment in high-fat-diet mice.[57] *Faecalibacteria* are known as beneficial butyrate producers in the human gut. A recent study found increased *Faecalibacterium prausnitzii* in patients treated with UDCA.[58] Llopis *et al.*[59] found decreased *Faecalibacterium* abundance in patients with ALD. In our study, we observed an increased abundance of *Roseburia*-related ASVs. The administration of *Roseburia* in a model of ALD resulted in the improvement of hepatic steatosis and inflammation,[60] suggesting a beneficial role of this strain in ALD.

In summary, we could demonstrate efficacy of norUDCA in experimental ALD, which might be caused by an increase in hepatic PPARg1 and enhanced macrophage polarisation towards an anti-inflammatory M2 phenotype. Future clinical trials for norUDCA in ALD are now warranted to prove its efficacy in ALDs in humans.

Abbreviations

Adh, alcohol dehydrogenase; ALD, alcohol-related liver disease; Aldh, aldehyde dehydrogenase; ALT, alanine transaminase; ARG, arginase; CLDN-4, claudin-4; CPT-1, carnitine palmitoyltransferase 1; Ctrl, control; CXCL-1, C-X-C motif chemokine ligand 1; CYP7A1, cytochrome P450 family 7 subfamily A member 1; cyto, cytosol; EtOH, ethanol; FASN, fatty acid synthase; FXR, farnesoid X receptor; GAPDH, glyceraldehyde-3-phosphate dehydrogenase; iNOS, inducible nitric oxide synthase; LBP, LPS-binding protein; LPS, lipopolysaccharide; MPO, myeloperoxidase; MRP4, multidrug resistance-associated protein 4; NAFLD, non-alcoholic fatty liver disease; NEMO, NF-κB essential modulator; NF-κB, nuclear factor kappa light chain enhancer of activated B cells; norUDCA, 24-norursodeoxycholic acid; nuc, nuclear extracts; PBMC, peripheral blood mononuclear cell; PPARg, peroxisome proliferator-activated receptor gamma; PSC, primary sclerosing cholangitis; SREBP1c, sterol regulatory element-binding protein 1c; SSU RNA, ribosomal small subunit; SULT2A1, sulfotransferase family 2A member 1; TJP-1, tight junction protein 1; TNF, tumour necrosis factor; TUNEL+, terminal deoxynucleotidyl transferase dUTP nick end labelling-positive; UDCA, ursodeoxycholic acid.

Financial support

HT is supported by the excellence initiative VASCage (Centre for Promoting Vascular Health in the Ageing Community), an R&D K-Centre (COMET program – Competence Centers for Excellent Technologies) funded by the Austrian Ministry for Transport, Innovation and Technology, the Austrian Ministry for Digital and Economic Affairs, and the federal states Tyrol, Salzburg, and Vienna. CG is supported by the Austrian Society of Gastroenterology and Hepatology (ÖGGH) and received speaker fees from Abbvie. TEA is supported by the Austrian Science Fund (FWF P33070) and by the European Union (ERC-STG Grant agreement No. 101039320). JS is supported by the Austrian Society of Gastroenterology and Hepatology (ÖGGH) and the German Society of Inflammatory Bowel Disease (DACED). MT is supported by the Austrian Science Fund (FWF) F7310. He received speaker fees from Falk Foundation, Gilead, Intercept, and MSD; he advised for Abbvie, Albireo, BiomX, Boehringer Ingelheim, Falk Pharma GmbH, Genfit, Gilead, Hightide, Intercept, Janssen, MSD, Novartis, Phenex, Pliant, Regulus, Siemens, and Shire. He further received travel grants from Abbvie, Falk, Gilead, and Intercept and research grants from Albireo, Alnylam, Cymabay, Falk, Gilead, Intercept, MSD, Takeda, and UltraGenyx. He is also the co-inventor of patents on the medical use of norUDCA filed by the Medical Universities of Graz and Vienna.

Conflicts of interest

The authors declare no conflict of interest.

Please refer to the accompanying ICMJE disclosure forms for further details.

Authors' contributions

Designed, performed, and analysed the animal and *in vitro* experiments together: CG, MM, SD, TC. Performed histologic analysis: GO. Performed microbiota analysis: BH, PP. Provided expertise and edited the manuscript: TEA, FG, JS. Prepared the manuscript: CG, MM. Coordinated the project: MT, HT.

Data availability statement

The data that support the findings of this study are available from the corresponding author upon reasonable request.

Acknowledgements

We thank Jasmin Schwarz and Gudrun Kohl for the help in sample processing for microbiome analysis.

Supplementary data

Supplementary data to this article can be found online at https://doi.org/10.1016/j.jhepr.2023.100872.

References

Author names in bold designate shared co-first authorship

[1] Rehm J, Samokhvalov AV, Shield KD. Global burden of alcoholic liver diseases. J Hepatol 2013;59:160–168.
[2] Avila MA, Dufour JF, Gerbes AL, Zoulim F, Bataller R, Burra P, et al. Recent advances in alcohol-related liver disease (ALD): summary of a Gut round table meeting. Gut 2020;69:764–780.
[3] **Haldar D**, **Kern B**, Hodson J, Armstrong MJ, Adam R, Berlakovich G, et al. Outcomes of liver transplantation for non-alcoholic steatohepatitis: a European Liver Transplant Registry study. J Hepatol 2019;71:313–322.
[4] Lieber CS. Alcoholic fatty liver: its pathogenesis and mechanism of progression to inflammation and fibrosis. Alcohol 2004;34:9–19.
[5] Yan AW, Fouts DE, Brandl J, Stärkel P, Torralba M, Schott E, et al. Enteric dysbiosis associated with a mouse model of alcoholic liver disease. Hepatology 2011;53:96–105.
[6] **Grander C**, **Adolph TE**, Wieser V, Lowe P, Wrzosek L, Gyongyosi B, et al. Recovery of ethanol-induced *Akkermansia muciniphila* depletion ameliorates alcoholic liver disease. Gut 2018;67:891–901.
[7] Hartmann P, Seebauer CT, Schnabl B. Alcoholic liver disease: the gut microbiome and liver cross talk. Alcohol Clin Exp Res 2015;39:763–775.
[8] Bajaj JS. Alcohol, liver disease and the gut microbiota. Nat Rev Gastroenterol Hepatol 2019;16:235–246.
[9] Lefevre AF, DeCarli LM, Lieber CS. Effect of ethanol on cholesterol and bile acid metabolism. J Lipid Res 1972;13:48–55.
[10] Vendemiale G, Jayatilleke E, Shaw S, Lieber CS. Depression of biliary glutathione excretion by chronic ethanol feeding in the rat. Life Sci 1984;34:1065–1073.
[11] Wu W, Zhu B, Peng X, Zhou M, Jia D, Gu J. Activation of farnesoid X receptor attenuates hepatic injury in a murine model of alcoholic liver disease. Biochem Biophys Res Commun 2014;443:68–73.
[12] Manley S, Ni HM, Williams JA, Kong B, DiTacchio L, Guo G, et al. Farnesoid X receptor regulates forkhead Box O3a activation in ethanol-induced autophagy and hepatotoxicity. Redox Biol 2014;2:991–1002.
[13] Xie G, Zhong W, Li H, Li Q, Qiu Y, Zheng X, et al. Alteration of bile acid metabolism in the rat induced by chronic ethanol consumption. FASEB J 2013;27:3583–3593.
[14] Manley S, Ding W. Role of farnesoid X receptor and bile acids in alcoholic liver disease. Acta Pharm Sin B 2015;5:158–167.
[15] Krenkel O, Tacke F. Liver macrophages in tissue homeostasis and disease. Nat Rev Immunol 2017;17:306–321.
[16] Tilg H, Moschen AR, Szabo G. Interleukin-1 and inflammasomes in alcoholic liver disease/acute alcoholic hepatitis and nonalcoholic fatty liver disease/nonalcoholic steatohepatitis. Hepatology 2016;64:955–965.
[17] Ceni E, Mello T, Galli A. Pathogenesis of alcoholic liver disease: role of oxidative metabolism. World J Gastroenterol 2014;20:17756–17772.
[18] **Fujisaka S**, **Usui I**, Nawaz A, Takikawa A, Kado T, Igarashi Y, et al. M2 macrophages in metabolism. Diabetol Int 2016;7:342–351.
[19] **Liu J**, **Geng X**, Hou J, Wu G. New insights into M1/M2 macrophages: key modulators in cancer progression. Cancer Cell Int 2021;21:389.
[20] **Wang C**, **Ma C**, Gong L, Guo Y, Fu K, Zhang Y, et al. Macrophage polarization and its role in liver disease. Front Immunol 2021;12:803037.
[21] Wu XQ, Yang Y, Li WX, Cheng YH, Li XF, Huang C, et al. Telomerase reverse transcriptase acts in a feedback loop with NF-κB pathway to regulate macrophage polarization in alcoholic liver disease. Sci Rep 2016;6:18685.
[22] Luo W, Xu Q, Wang Q, Wu H, Hua J. Effect of modulation of PPAR-γ activity on Kupffer cells M1/M2 polarization in the development of non-alcoholic fatty liver disease. Sci Rep 2017;7:44612.
[23] Bouhlel MA, Derudas B, Rigamonti E, Dièvart R, Brozek J, Haulon S, et al. PPARγ activation primes human monocytes into alternative M2 macrophages with anti-inflammatory properties. Cell Metab 2007;6:137–143.
[24] Straus DS, Glass CK. Anti-inflammatory actions of PPAR ligands: new insights on cellular and molecular mechanisms. Trends Immunol 2007;28:551–558.
[25] Halilbasic E, Fiorotto R, Fickert P, Marschall HU, Moustafa T, Spirli C, et al. Side chain structure determines unique physiologic and therapeutic properties of norursodeoxycholic acid in *Mdr2*$^{-/-}$ mice. Hepatology 2009;49:1972–1981.
[26] Denk GU, Maitz S, Wimmer R, Rust C, Invernizzi P, Ferdinandusse S, et al. Conjugation is essential for the anticholestatic effect of NorUrsodeoxycholic acid in taurolithocholic acid-induced cholestasis in rat liver. Hepatology 2010;52:1758–1768.
[27] Beraza N, Ofner-Ziegenfuss L, Ehedego H, Boekschoten M, Bischoff SC, Mueller M, et al. Nor-ursodeoxycholic acid reverses hepatocyte-specific NEMO-dependent steatohepatitis. Gut 2011;60:387–396.
[28] Sombetzki M, Fuchs CD, Fickert P, Österreicher CH, Mueller M, Claudel T, et al. 24-nor-ursodeoxycholic acid ameliorates inflammatory response and liver fibrosis in a murine model of hepatic schistosomiasis. J Hepato 2015;62:871–878.
[29] Trauner M, Fuchs CD, Halilbasic E, Paumgartner G. New therapeutic concepts in bile acid transport and signaling for management of cholestasis. Hepatology 2017;65:1393–1404.
[30] Zhu C, Boucheron N, Müller AC, Májek P, Claudel T, Halilbasic E, et al. 24-Norursodeoxycholic acid reshapes immunometabolism in CD8^{+} T cells and alleviates hepatic inflammation. J Hepatol 2021;75:1164–1176.
[31] Fickert P, Hirschfield GM, Denk G, Marschall HU, Altorjay I, Färkkilä M, et al. norUrsodeoxycholic acid improves cholestasis in primary sclerosing cholangitis. J Hepatol 2017;67:549–558.
[32] Traussnigg S, Schattenberg JM, Demir M, Wiegand J, Geier A, Teuber G, et al. Norursodeoxycholic acid versus placebo in the treatment of non-alcoholic fatty liver disease: a double-blind, randomised, placebo-controlled, phase 2 dose-finding trial. Lancet Gastroenterol Hepatol 2019;4:781–793.
[33] Feldman AT, Wolfe D. Tissue processing and hematoxylin and eosin staining. Methods Mol Biol 2014;1180:31–43.
[34] Herbold CW, Pelikan C, Kuzyk O, Hausmann B, Angel R, Berry D, et al. A flexible and economical barcoding approach for highly multiplexed amplicon sequencing of diverse target genes. Front Microbiol 2015;6:731.
[35] Apprill A, McNally S, Parsons R, Weber L. Minor revision to V4 region SSU rRNA 806R gene primer greatly increases detection of SAR11 bacterioplankton. Aquat Microb Ecol 2015;75:129–137.
[36] Brigo N, Pfeifhofer-Obermair C, Tymoszuk P, Demetz E, Engl S, Barros-Pinkelnig M, et al. Cytokine-mediated regulation of ARG1 in macrophages and its impact on the control of *Salmonella enterica* Serovar Typhimurium infection. Cells 2021;10:1823.
[37] **Grander C**, **Schaefer B**, **Schwärzler J**, Grabherr F, de Graaf DM, Enrich B, et al. Alpha-1 antitrypsin governs alcohol-related liver disease in mice and humans. Gut 2021;70:585–594.
[38] Younossi ZM, Blissett D, Blissett R, Henry L, Stepanova M, Younossi Y, et al. The economic and clinical burden of nonalcoholic fatty liver disease in the United States and Europe. Hepatology 2016;64:1577–1586.
[39] Bajaj JS, Heuman DM, Hylemon PB, Sanyal AJ, White MB, Monteith P, et al. Altered profile of human gut microbiome is associated with cirrhosis and its complications. J Hepatol 2014;60:940–947.
[40] Ju C, Mandrekar P. Macrophages and alcohol-related liver inflammation. Alcohol Res 2015;37:251–262.
[41] Sica A, Mantovani A. Macrophage plasticity and polarization: in vivo veritas. J Clin Invest 2012;122:787–795.
[42] Louvet A, Teixeira-Clerc F, Chobert MN, Deveaux V, Pavoine C, Zimmer A, et al. Cannabinoid CB2 receptors protect against alcoholic liver disease by regulating Kupffer cell polarization in mice. Hepatology 2011;54:1217–1226.
[43] Fajas L, Auboeuf D, Raspé E, Schoonjans K, Lefebvre AM, Saladin R, et al. The organization, promoter analysis, and expression of the human PPARγ gene. J Biol Chem 1997;272:18779–18789.
[44] Welch JS, Ricote M, Akiyama TE, Gonzalez FJ, Glass CK. PPARγ and PPARδ negatively regulate specific subsets of lipopolysaccharide and IFN-γ target genes in macrophages. Proc Natl Acad Sci U S A 2003;100:6712–6717.
[45] Strand DW, Jiang M, Murphy TA, Yi Y, Konvinse KC, Franco OE, et al. PPARγ isoforms differentially regulate metabolic networks to mediate mouse prostatic epithelial differentiation. Cell Death Dis 2012;3:e361.
[46] Lee YK, Park JE, Lee M, Hardwick JP. Hepatic lipid homeostasis by peroxisome proliferator-activated receptor gamma 2. Liver Res 2018;2:209–215.
[47] Ratziu V, Charlotte F, Bernhardt C, Giral P, Halbron M, Lenaour G, et al. Long-term efficacy of rosiglitazone in nonalcoholic steatohepatitis: results of the Fatty Liver Improvement by Rosiglitazone Therapy (FLIRT 2) extension trial. Hepatology 2010;51:445–453.
[48] García-Ruiz I, Rodríguez-Juan C, Díaz-Sanjuán T, Martínez MA, Muñoz-Yagüe T, Solís-Herruzo JA. Effects of rosiglitazone on the liver histology and mitochondrial function in ob/ob mice. Hepatology 2007;46:414–423.
[49] Torres DM, Jones FJ, Shaw JC, Williams CD, Ward JA, Harrison SA. Rosiglitazone versus rosiglitazone and metformin versus rosiglitazone and losartan in the treatment of nonalcoholic steatohepatitis in humans: a 12-month randomized, prospective, open- label trial. Hepatology 2011;54:1631–1639.
[50] Ratziu V, Giral P, Jacqueminet S, Charlotte F, Hartemann-Heurtier A, Serfaty L, et al. Rosiglitazone for nonalcoholic steatohepatitis: one-year

results of the randomized placebo-controlled fatty liver improvement with rosiglitazone therapy (FLIRT) trial. Gastroenterology 2008;135:100–110.

[51] Yu JH, Song SJ, Kim A, Choi Y, Seok JW, Kim HJ, et al. Suppression of PPARγ-mediated monoacylglycerol O-acyltransferase 1 expression ameliorates alcoholic hepatic steatosis. Sci Rep 2016;6:29352.

[52] Zhang W, Sun Q, Zhong W, Sun X, Zhou Z. Hepatic peroxisome proliferator-activated receptor gamma signaling contributes to alcohol-induced hepatic steatosis and inflammation in mice. Alcohol Clin Exp Res 2016;40:988–999.

[53] Schwandt ML, Diazgranados N, Umhau JC, Kwako LE, George DT, Heilig M. PPARγ activation by pioglitazone does not suppress cravings for alcohol, and is associated with a risk of myopathy in treatment seeking alcohol dependent patients: a randomized controlled proof of principle study. Psychopharmacology 2020;237:2367–2380.

[54] Thieringer R, Fenyk-Melody JE, Le Grand CB, Shelton BA, Detmers PA, Somers EP, et al. Activation of peroxisome proliferator-activated receptor γ does not inhibit IL-6 or TNF-α responses of macrophages to lipopolysaccharide in vitro or in vivo. J Immunol 2000;164:1046–1054.

[55] Gao S, Zhou J, Liu N, Wang L, Gao Q, Wu Y, et al. Curcumin induces M2 macrophage polarization by secretion IL-4 and/or IL-13. J Mol Cell Cardiol 2015;85:131–139.

[56] Wagner MC, Yeligar SM, Brown LA, Michael Hart C. PPARγ ligands regulate NADPH oxidase, eNOS, and barrier function in the lung following chronic alcohol ingestion. Alcohol, Clin Exp Res 2012;36:197–206.

[57] Li H, Wang Q, Chen P, Zhou C, Zhang X, Chen L. Ursodeoxycholic acid treatment restores gut microbiota and alleviates liver inflammation in non-alcoholic steatohepatitic mouse model. Front Pharmacol 2021;12:788558.

[58] Pearson T, Caporaso JG, Yellowhair M, Bokulich NA, Padi M, Roe DJ, et al. Effects of ursodeoxycholic acid on the gut microbiome and colorectal adenoma development. Cancer Med 2019;8:617–628.

[59] Llopis M, Cassard AM, Wrzosek L, Boschat L, Bruneau A, Ferrere G, et al. Intestinal microbiota contributes to individual susceptibility to alcoholic liver disease. Gut 2015.

[60] Seo B, Jeon K, Moon S, Lee K, Kim WK, Jeong H, et al. *Roseburia* spp. abundance associates with alcohol consumption in humans and its administration ameliorates alcoholic fatty liver in mice. Cell Host Microbe 2020;27:25–40.e26.

Journal of Hepatology, Volume *5*

Supplemental information

24-Norursodeoxycholic acid ameliorates experimental alcohol-related liver disease and activates hepatic PPARγ

Christoph Grander, Moritz Meyer, Daniel Steinacher, Thierry Claudel, Bela Hausmann, Petra Pjevac, Felix Grabherr, Georg Oberhuber, Manuel Grander, Natascha Brigo, Almina Jukic, Julian Schwärzler, Günter Weiss, Timon E. Adolph, Michael Trauner, and Herbert Tilg

24-Norursodeoxycholic acid ameliorates experimental alcohol-related liver disease and activates hepatic PPARγ

C. Grander, M. Meyer D. Steinacher, T. Claudel, B. Hausmann, P. Pjevac, F. Grabherr, G. Oberhuber, M. Grander, N. Brigo, A. Jukic, J. Schwärzler, G. Weiss, T.E. Adolph, M. Trauner, H. Tilg

Table of contents

Supplementary materials and methods 2

Supplementary figures.. 9

Supplementary references.. 15

Supplementary materials and methods

Immunohistochemistry and TUNEL labelling

Liver sections were deparaffinised in xylene and dehydrated in an ethanol gradient. Antigen unmasking was performed by using a 2 % citrate-buffer (pH = 6, Vector Laboratories, Burlingame, USA) in a conventional steamer. Inactivation of endogenous peroxidase activity was carried out by using Peroxidase Blocking Solution (Dako, Santa Clara, CA, USA), protein blocking was performed with a ready-to-use kit (MP-740; Dako, Santa Clara, CA, USA). Afterwards, antibodies (MPO, Dako, Santa Clara, CA, USA; F4/80, Cell Signaling Technology #70076) and secondary anti-rabbit antibodies (Dako, Santa Clara, CA, USA) were applied. To visualize immunoreactivity, ImmPACT AMEC (Vector Laboratories, Burlingame, CA, USA) was used, followed by staining with DAB (Dako, Santa Clara, CA, USA) for 2 minutes and counterstaining with hematoxylin (Dako, Santa Clara, CA, USA) for 20 seconds. MPO^+ and $F4/80^+$ cells were counted in 10 randomly selected fields of $1mm^2$. TUNEL labeling was performed according to the manufacturer's instructions (Roche, 11684817910). $TUNEL^+$ cells were quantified in 10 randomly selected fields of $1mm^2$ by counting TUNEL+ cells per HPF.

Triglyceride measurement

Frozen liver tissue samples were homogenized in PBS (volume was adjusted to the liver tissue weight) followed by an incubation at 60° C for 30 minutes. Afterwards, samples were centrifuged (12.000 G, 10 min, room temperature) and supernatant was taken off. Triglyceride concentration was measured with TG-Reagent (Roche, Basel, Switzerland) according to the manufacturer instructions.

Ethanol measurement

Ethanol concentration in serum samples was measured using the EnzyChrom assay (ECET-100, BioAssay Systems, Hayward, CA, USA) according to the manufacturer instructions.

Western blot

Hepatic protein was isolated using T-PER tissue protein extraction reagent. The reagent was supplemented with HALT proteinase inhibitor cocktail (Thermo Fisher Scientific, Waltham, MA, USA). Protein concentrations were measured by Bradford Protein Assay (BioRad, 5000006), separated by SDS-PAGE (Hercules, Bio Rad, CA, USA) and blotted onto Hybond-P PVDF membranes (GE Healthcare, Chicago, IL, USA). Blocking of membranes was performed with 5% skim milk. Membranes were incubated overnight with the primary antibody. Following antibodies were used for detection: PPARg, Cell Signaling Technology #2443; PPARg A3409A, Invitrogen; CPT1A, Cell Signaling Technology #97361; GAPDH, Cell Signaling Technology #2118. Visualization of immunoreactivity was carried out by using HRP-conjugated secondary antibodies (Cell Signalling Technology, #7074) and ECL Select Western Blotting Detection Reagent (Amersham, RPN2235). GAPDH (GAPDH, glyceraldehyde 3-phosphate dehydrogenase) was used as a reference protein. Quantitation of the western blot signals was performed using the Biorad ChemiDoc MP (Hercules, CA, USA). Densitometry of immunoblots was performed with BioRad Image Lab software.

FACS Analysis:

Bone marrow derived macrophages (BMDM) from C57BL/6N mice were prepared as described (Brigo et al. 10.21769/BioProtoc.4440). After 5 days of differentiation with 50 ng/ml macrophage colony stimulating factor (MCSF, Peprotech), cells were subjected to lipopolysaccharide (LPS) (100 ng/mL; Invivogen, San Diego, CA, USA), Interferon-γ for M1 polarization (100 ng/mL; Peprotech, London, United Kingdom) or Interleukin 4 for M2 polarization (10ng/ml; Peprotech, London, United Kingdom) or norUCDA stimulation for 24h. The following day, cells were scraped and stained for FACS analysis as previously described [1]. Percentage of M1 (iNOS+; CD80+) or M2 (Arg1+; CD206+) macrophages was determined by flow cytometry analysis using a CytoFLEX S (Beckman Coulter). The gating strategy is shown in Fig. S2. Bars indicates mean +- SEM.

ELISA:

IL-6 concentration was measured using commercially available ELISA kits from R&D systems (Minneapolis, MN, USA) according to the manufacturer's Instructions.

Microbiome-Studies:

The obtained sequence data was quality-filtered and demultiplexed, followed by amplicon sequencing variant (ASV) inference with DADA2 [2](4), enabling analysis at the highest possible taxonomic resolution. Resulting ASV sequences were taxonomically classified using SINA [3] with the newest release of the the SILVA SSU rRNA database [4]. If necessary, contaminants were removed in silico using the decontam software package [5].

Abundance measurements (counts) of ASVs, as well as ASV sum counts at higher taxonomic levels were statistically evaluated, to test for significant differences in microbial community composition between the subject groups. Detection of significantly more abundant amplicon sequence variants in the case over the controls weas performed, and adjusted P-values were calculated using the Benjamini-Hochberg method and differences supported with P-values < 0.05 were considered significant.

Statistical analysis was performed with the metagenomeSeq software, which has been proven optimal for amplicon datasets [6]. MetagenomeSeq normalized the abundance data to address varying depths of sequencing coverage across samples, and then a zero-inflated log-normal mixture model was applied to calculated the fold changes between the case and control group for each taxonomic level [7].

<u>In-vitro studies:</u>

Inflammation analysis: Peripheral Blood Mononuclear Cells (PBMCs) were collected from five healthy volunteers. Blood samples were collected in heparinized tubes, followed by density gradient centrifugation of whole blood samples on Lymphoprep solution according to the manufacturer’s instructions (Axis Shields, Oslo, Norway) [8]. After isolation, PBMCs were stimulated with lipopolysaccharide (LPS) (1 pg/mL; Invivogen, San Diego, CA, USA) for 24 hours and 50µM as well as 500µM norUDCA. DMSO served as negative control. 24 hours after stimulation, cells supernatant was harvested and IL-6 was measured by ELISA.

Cell culture and luciferase assay:

Human primary immortalized hepatocytes (IHH) [9] were adapted to DMEM with 10% fetal bovine serum (FBS) and 1% penicillin/streptomycin (all from Thermo Fischer, Vienna, Austria). IHH were seeded in a 24 well-plate, then transiently transfected with 150 ng/well of PPARg response element (located into the promoter of PPARg [10] cloned in 3 copies in front of a minimum thymidine kinase promoter linked to a luciferase (TK-luc-pGl3) construct and 100ng of pSG5-PPARg2 expressing plasmid (a gift from Dr Johan Auwerx, EPFL Lausanne, Switzerland) using Fugene transfection reagent (Promega, Madison, WI) in sterile DMEM without FBS for 12 h. Medium containing 10% FBS was then added for 24 h with NorUDCA 500 µM and the cells lysed using a lysis solution (4% Triton-X100, Glycyl-Glycine 100mM, $MgSO_4$ 100mM, EGTA 250mM) for 1 h at room temperature on a shaker. Cell extracts were then combined with the luciferin solution (Luciferin 2.5mM and ATP 20mM, Merck, Vienna) and analyzed with a luminometer (Lumat LB9507 EG&G Berthold, Germany).

Electrophoretic mobility shift assay (EMSA):

IHH cells were cultivated for 48 h with or without NorUDCA 500µM or rosiglitazone 10µM (Merck, Vienna, Austria) in DMEM without fetal calf serum, with 0.5 % penicillin/streptomycin (ThermoFisher Scientific, Vienna, Austria) and nuclear extracts (NE) were prepared. Briefly, cells were centrifuged for 5 min at 1,000 rpm, the pellet dissolved in homogenization buffer (15mM Tris HCl, pH 8; 15mM NaCl, 60mM KCl; 0.5mM EDTA; 1mM PMSF and 1mM beta mercaptoethanol). After centrifugation during 5 min at 1,000 rpm, cell pellets were suspended in Hypotonic Buffer (HB) containing 0.05 % Triton X-100. After another centrifugation at 1,200 rpm the supernatant (cytosolic fractions) were collected and kept at -80°C. The pellets were

washed with 5 ml of HB buffer with Triton and centrifuged for 10 min at 1,200 rpm, the pellet was washed with 5 ml of HB buffer without Triton and centrifuged at 1,200 rpm for 10 min. Finally, pellets were suspended in 50 µl of HB buffer modified with 360mM KCl and incubated at 4°C for 30 min, before a final centrifugation for 5 min at 13,000 rpm. The supernatants (nuclear fractions) were kept at -80°C before use in gel shift. Protein concentrations were measured by the bicinchoninic assay (ThermoFisher, Vienna, Austria). Double-stranded oligonucleotides (sequence 5′-GATCCTAGAATATAGGTCAGGGAAG-3) were end labeled with γ-32P ATP (Hartmann Analytic, Braunschweig, Germany) using T4- polynucleotide kinase (New England Biolabs, Frankfurt am Main, Germany) and purified by column elution using the QIAquick nucleotide removal kit (Qiagen, Hilden, Germany). Oligonucleotides and cytosolic and nuclear extracts (1 µg) were mixed in a DNA binding buffer containing in a 20µl final volume (10mM Tris pH 7.5, 150mM NaCl, 1 mMDTT, 1mM EDTA, 5% glycerol (Sigma-Aldrich, Vienna, Austria), for 10 minutes at room temperature before the radiolabeled probe (0.5 ng) was added. Binding reactions were further incubated for 10 minutes with PPARg antibody (sc-7273X, Santa-Cruz biotechnology, Heidelberg, Germany) and resolved by 4 % non-denaturing polyacrylamide gel electrophoresis in 0.25X Tris-Borate-EDTA (TBE) buffer at room temperature. After run, gel was dried for 1h at 60°C and transferred into developing cassette (Biomax, Kodak) for overnight film exposure at -80C.

Quantification of alanine aminotransferase (ALT) in mouse serum

The quantification of murine ALT (alanine aminotransferase) in serum samples was performed by using an enzymatic assay (BQ-Kit, San Diego, CA, USA) according to the manufacturer instructions.

Culture and stimulation of HepG2

HepG2s were cultured in RPMI (supplemented with 10% fetal calf serum, Sigma, St. Louis, MO, USA) as described above [11]. Cells were stimulated with recombinant human IL-6 (10 ng/ml; Peprotech Austria, Vienna, Austria), recombinant IL-1β (1 ng/ml, Peprotech Austria, Vienna, Austria) or lipopolysaccharide (LPS) (100 ng/mL; Invivogen, San Diego, CA, USA) for four and 24 hours. As negative control cells were stimulated with DMSO. Four and 24 hours after stimulation, cells were harvested and RNA was extracted and further processed as described above.

RNA isolation and PCR of liver tissue

Tissue samples were homogenized in TRIzol (Thermo Fisher Scientific, Waltham, MA, USA) using a metal bead homogenizing system (Precellys, Bertin Technologies, Montigny-le-Bretonneux, France) followed by RNA isolation. Reverse transcription was accomplished with Reverse Transcription System (Thermo Fisher Scientific, Waltham, MA, USA), followed by quantitative real-time PCR using SybrGreen (Eurogentec, Seraing, Belgium) and the Mx3000 qPCR cycler (Stratagene California, San Diego, CA, USA). βActin was used as a reference gene. All used PCR primers are available on request.

Supplementary figures

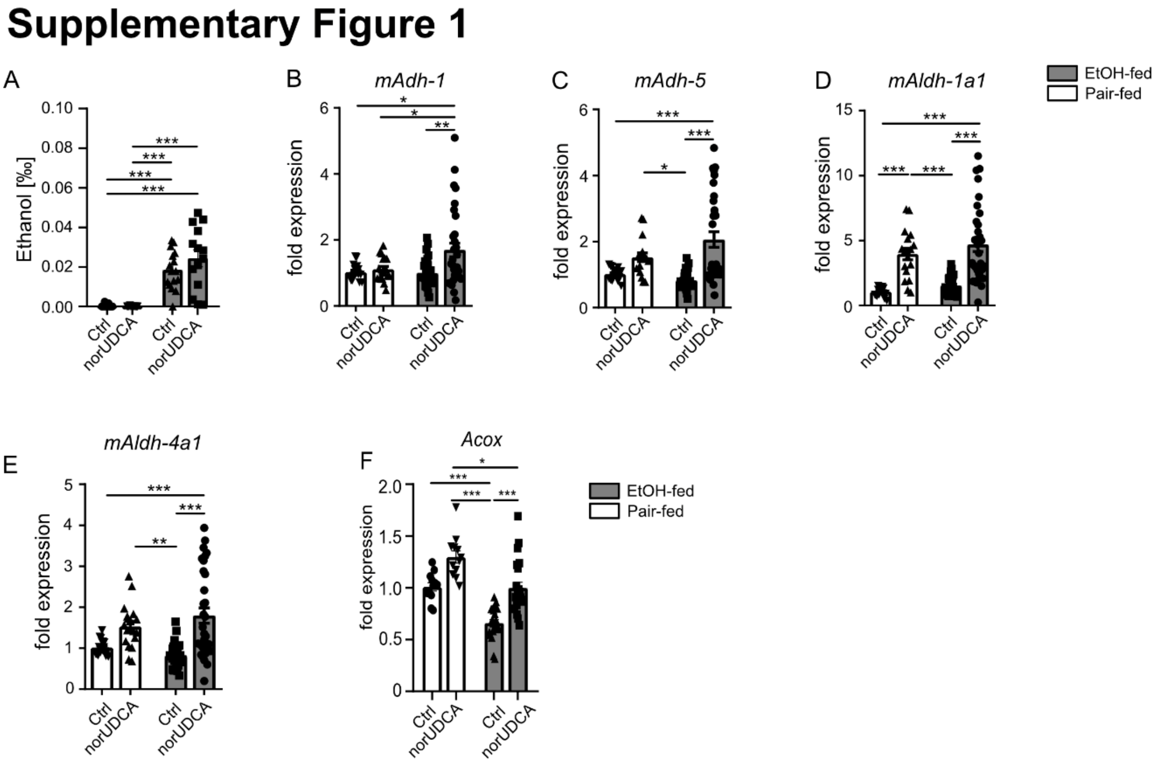

Fig. S1: (A) Shown is the serum concentration of ethanol. Data are shown as mean ± SEM. *p<0.05; **p<0.01; ***p<0.001 according to one-way ANOVA with Bonferroni post-hoc analysis. Acox, peroxisomal acyl-coenzyme A oxidase 1; Adh, alcohol-dehydrogenase; aldh, aldehyde- dehydrogenase; Ctrl, control; EtOH, ethanol.

Supplementary Figure 2

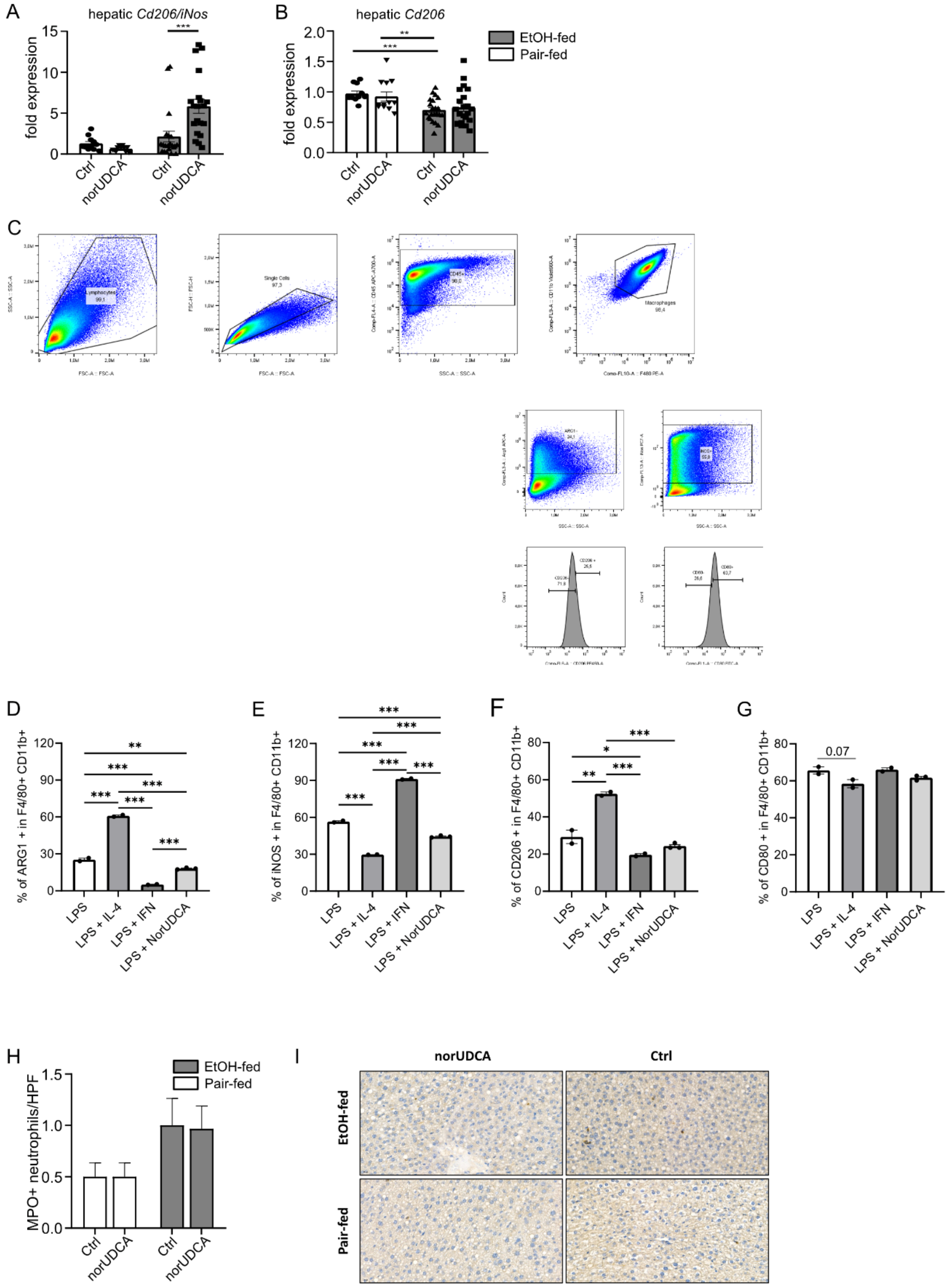

Fig. S2: (A) Shown is the hepatic mRNA expression ratio of *Cd206/iNos* (B) and *Cd206*. (C) Bone marrow derived macrophages were stimulated with 100 ng/ml LPS;

100 ng/ml IFNγ; 10 ng/ml IL-4 and 500μM norUDCA. After 24h of stimulation BMDM were scraped, stained and analyzed with FACS. Gating strategy for single cells, CD45+, CD11b+ F4/80+ double positive cells. To differentiate between M1 and M2 macrophages, iNOS+ and CD80+ (M1) cells as well as ARG1+ and CD206+ (M2) cells were determined. Quantification of ARG1+ (D), iNOS+ (E), CD206+ (F), CD80+ (G) BMDMs, determined by flow cytometry and presented in percent of F4/80+CD11b+ single cells indicating macrophage polarization towards M1 (E, G) and M2 (D, F) phenotype. (H) Number of MPO^+ cells was unchanged after norUDCA treatment in ethanol-fed mice compared to controls. (I) Representative images of MPO stained liver sections (brown indicates MPO^+ cells, black arrow). Data are shown as mean ± SEM. *p<0.05; **p<0.01; ***p<0.001 according to one-way ANOVA with Bonferroni post-hoc analysis or Kruskal-Wallis test with Dunn's post-hoc analysis. β-Actin was used as a house-keeping gene (A-B). Ctrl, control; EtOH, ethanol; LPS, lipopolysaccharide; iNOS, Inducible nitric oxide synthase; IL, interleukin; IFN, interferon; MPO, myeloperoxidase.

Supplementary Figure 3

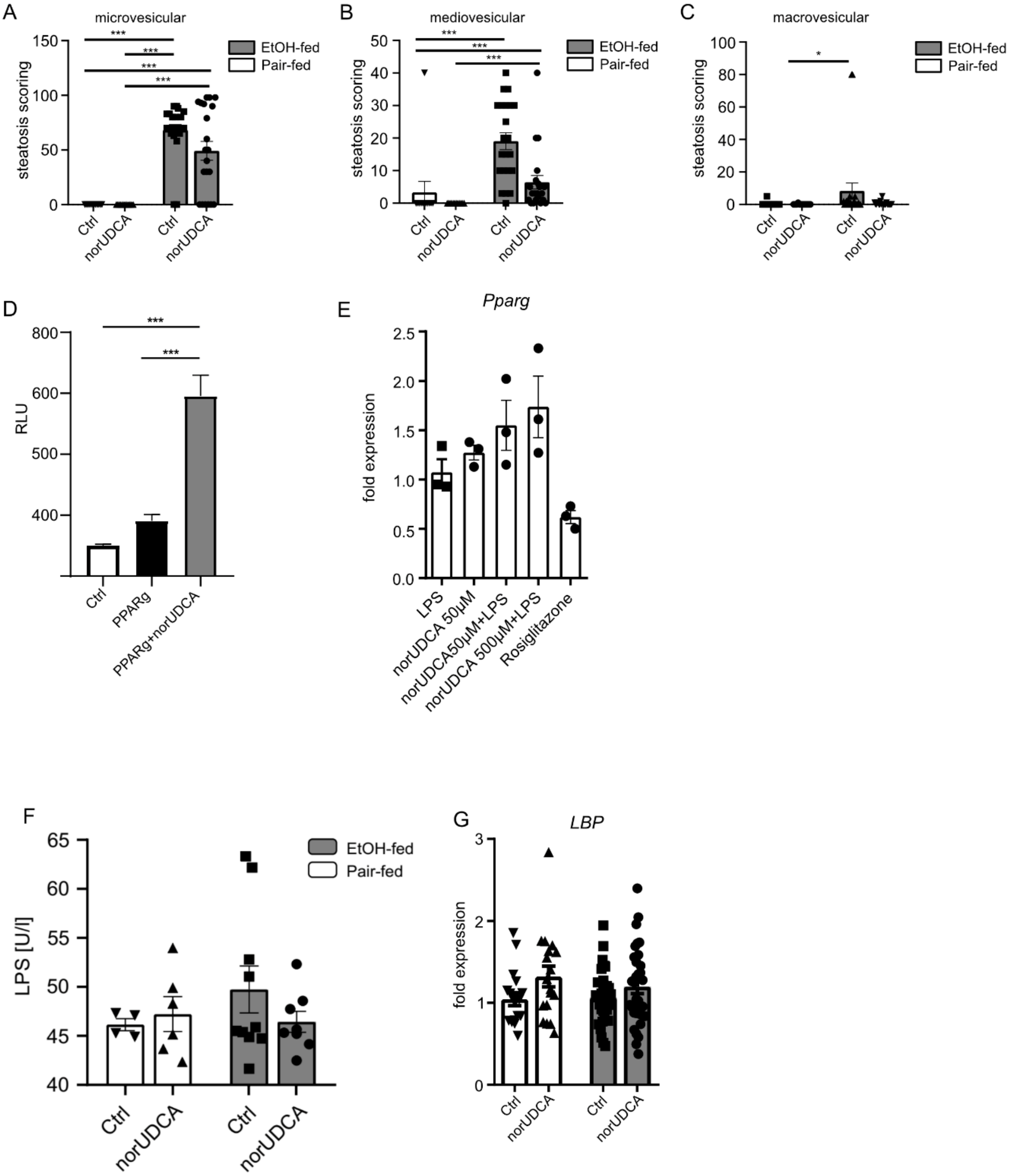

Fig. S3:

Staging of hepatic steatosis based on (A) microvesicular (B) mediovesicular (C) macrovesicular steatosis. (D) Luciferase assay with human primary immortalized hepatocytes was used to test PPAR-gamma activation upon norUDCA stimulation. (E)

Pparg mRNA expression after treatment of HepG2 cells with norUDCA and LPS compared to house-keeping gene ß-actin. (F) Serum LPS concentration of ethanol- and Pair-fed mice with and without norUDCA treatment. (G) LPS-binding protein (LBP) mRNA expression is not significantly altered between the groups. Data are shown as mean ± SEM. *p<0.05; **p<0.01; ***p<0.001 according to one-way ANOVA with Bonferroni post-hoc analysis or Kruskal-Wallis test with Dunn's post-hoc analysis. β-Actin was used as a house-keeping gene (E, G). Ctrl, control; EtOH, ethanol; LBP, LPS binding protein; LPS, lipopolysaccharide; Pparg, peroxisome proliferator-activated receptor gamma.

Suppl Figure 4

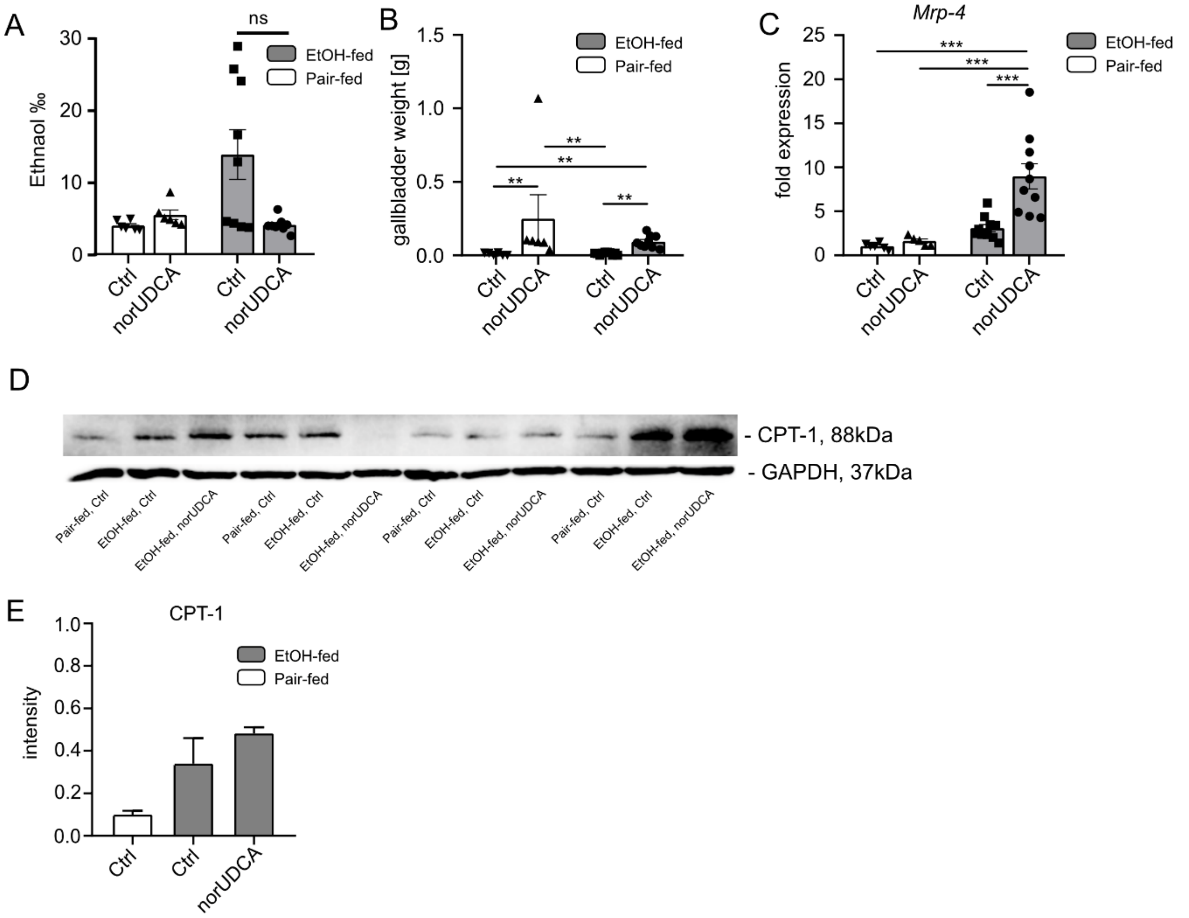

Fig. S4:

(A) Shown is the serum concentration of ethanol. (B) Gallbladder weight was increased upon norUDCA treatment. (C) Hepatic expression of *Mrp-4.* (D) Westernblot analysis of CPT-1 with GAPDH with (E) quantification of CPT-1. Data are shown as mean ± SEM. *p<0.05; **p<0.01; ***p<0.001 according to one-way ANOVA with Bonferroni post-hoc analysis or Kruskal-Wallis test with Dunn's post-hoc analysis. β-Actin was used as a house-keeping gene (C). Ctrl, control; EtOH, ethanol, Cpt-1, Carnitine palmitoyltransferase 1; GAPDH, Glyceraldehyde-3-phosphate dehydrogenase, MRP-4, multidrug resistance-associated protein 4.

Supplementary references

[1] Brigo N, Pfeifhofer-Obermair C, Tymoszuk P, Demetz E, Engl S, Barros-Pinkelnig M, et al. Cytokine-Mediated Regulation of ARG1 in Macrophages and Its Impact on the Control of Salmonella enterica Serovar Typhimurium Infection. Cells 2021;10.

[2] Callahan BJ, McMurdie PJ, Rosen MJ, Han AW, Johnson AJ, Holmes SP. DADA2: High-resolution sample inference from Illumina amplicon data. Nature methods 2016;13:581-583.

[3] Pruesse E, Peplies J, Glöckner FO. SINA: accurate high-throughput multiple sequence alignment of ribosomal RNA genes. Bioinformatics (Oxford, England) 2012;28:1823-1829.

[4] Quast C, Pruesse E, Yilmaz P, Gerken J, Schweer T, Yarza P, et al. The SILVA ribosomal RNA gene database project: improved data processing and web-based tools. Nucleic acids research 2013;41:D590-596.

[5] Davis NM, Proctor DM, Holmes SP, Relman DA, Callahan BJ. Simple statistical identification and removal of contaminant sequences in marker-gene and metagenomics data. Microbiome 2018;6:226.

[6] Thorsen J, Brejnrod A, Mortensen M, Rasmussen MA, Stokholm J, Al-Soud WA, et al. Large-scale benchmarking reveals false discoveries and count transformation sensitivity in 16S rRNA gene amplicon data analysis methods used in microbiome studies. Microbiome 2016;4:62.

[7] Paulson JN, Stine OC, Bravo HC, Pop M. Differential abundance analysis for microbial marker-gene surveys. Nature methods 2013;10:1200-1202.

[8] Moschen AR, Geiger S, Krehan I, Kaser A, Tilg H. Interferon-alpha controls IL-17 expression in vitro and in vivo. Immunobiology 2008;213:779-787.

[9] Schippers IJ, Moshage H, Roelofsen H, Müller M, Heymans HS, Ruiters M, et al. Immortalized human hepatocytes as a tool for the study of hepatocytic (de-)differentiation. Cell Biol Toxicol 1997;13:375-386.

[10] Gervois P, Chopin-Delannoy S, Fadel A, Dubois G, Kosykh V, Fruchart JC, et al. Fibrates increase human REV-ERBalpha expression in liver via a novel peroxisome proliferator-activated receptor response element. Mol Endocrinol 1999;13:400-409.

[11] Ress C, Moschen AR, Sausgruber N, Tschoner A, Graziadei I, Weiss H, et al. The role of apolipoprotein A5 in non-alcoholic fatty liver disease. Gut 2011;60:985-991.
